# Private measures, random walks, and synthetic data

**DOI:** 10.1007/s00440-024-01279-z

**Published:** 2024-04-20

**Authors:** March Boedihardjo, Thomas Strohmer, Roman Vershynin

**Affiliations:** 1 Department of Mathematics, Michigan State University, East Lansing, USA; 2 Department of Mathematics, University of California, Davis, Davis, USA; 3 Department of Mathematics, University of California, Irvine, Irvine, USA

**Keywords:** Random walks, Differential privacy, Synthetic data, 60G50, 68P27

## Abstract

Differential privacy is a mathematical concept that provides an information-theoretic security guarantee. While differential privacy has emerged as a de facto standard for guaranteeing privacy in data sharing, the known mechanisms to achieve it come with some serious limitations. Utility guarantees are usually provided only for a fixed, a priori specified set of queries. Moreover, there are no utility guarantees for more complex—but very common—machine learning tasks such as clustering or classification. In this paper we overcome some of these limitations. Working with metric privacy, a powerful generalization of differential privacy, we develop a polynomial-time algorithm that creates a *private measure* from a data set. This private measure allows us to efficiently construct private synthetic data that are accurate for a wide range of statistical analysis tools. Moreover, we prove an asymptotically sharp min-max result for private measures and synthetic data in general compact metric spaces, for any fixed privacy budget $\varepsilon$ bounded away from zero. A key ingredient in our construction is a new *superregular random walk*, whose joint distribution of steps is as regular as that of independent random variables, yet which deviates from the origin logarithmically slowly.

## Introduction

### Motivation

The right to privacy is enshrined in the Universal Declaration of Human Rights [45]. However, as artificial intelligence is more and more permeating our daily lives, data sharing is increasingly locking horns with data privacy concerns. Differential privacy (DP), a probabilistic mechanism that provides an information-theoretic privacy guarantee, has emerged as a de facto standard for implementing privacy in data sharing [22]. For instance, DP has been adopted by several tech companies [20] and will also be used in connection with the release of the Census 2020 data [2, 3].

Yet, current embodiments of DP come with some serious limitations [17, 25, 53]:

(i) Utility guarantees are usually provided only for a fixed set of queries. This means that either DP has to be used in an interactive scenario or the queries have to specified in advance.
(ii) There are no utility guarantees for more complex—but very common—machine learning tasks such as clustering or classification.
(iii) DP can suffer from a poor privacy-utility tradeoff, leading to either insufficient privacy protection or to data sets of rather low utility, thereby making DP of limited use in many applications [17].

Another approach to enable privacy in data sharing is based on the concept of synthetic data [8]. The goal of synthetic data is to create a dataset that maintains the statistical properties of the original data while not exposing sensitive information. The combination of differential privacy with synthetic data has been suggested as a best-of-both-world solutions [8, 12, 23, 30, 34]. While combining DP with synthetic data can indeed provide more flexibility and thereby partially address some of the issues in (i), in and of itself it is not a panacea for the aforementioned problems.

One possibility to construct differentially private synthetic datasets that are not tailored to a priori specified queries is to simply add independent Laplacian noise to each data point. However, the amount noise that has to be added to achieve sufficient DP is too large with respect to maintaining satisfactory utility even for basic counting queries [54], not to mention more sophisticated machine learning tasks.

This raises the fundamental question whether it is even possible to construct in a numerically efficient manner differentially private synthetic data that come with rigorous utility guarantees for a wide range of (possibly complex) queries, while achieving a favorable privacy-utility tradeoff? In this paper we will answer this question to the affirmative.

### A private measure

A main objective of this paper is to construct a *private measure* on a given metric space $(T,\rho )$. Namely, we design an algorithm that transforms a probability measure $\mu$ on *T* into another probability measure $\nu$ on *T*, and such that this transformation is both private and accurate.

For clarity, let us first consider the special case of empirical measures, where our goal can be understood as creating *differentially private synthetic data*. Specifically, we are looking for a computationally tractable algorithm that transforms true input data $X =(X_1,\ldots ,X_n) \in T^n$ into synthetic output data $Y=(Y_1,\ldots ,Y_m) \in T^m$ for some *m*, and which is $\varepsilon$-differentially private (see Definition 2.1) and such that the empirical measures

$$\begin{aligned} \mu _X = \frac{1}{n} \sum _{i=1}^n \delta _{X_i} \quad \text {and} \quad \mu _Y = \frac{1}{m} \sum _{i=1}^m \delta _{Y_i} \end{aligned}$$

are close to each other in the Wasserstein 1-metric (recalled in Sect. 2.2.2):

1.1 $$\begin{aligned} {{\,\mathrm{{\mathbb {E}}}\,}}W_1 \left( \mu _X,\mu _Y \right) \le \gamma , \end{aligned}$$

where $\gamma >0$ is as small as possible. In other words, our goal is to create synthetic data *Y* from the true data *X* by adding noise of average magnitude $\gamma$, just not necessarily i.i.d. noise.

The main result of this paper is a computationally effective private algorithm whose accuracy $\gamma$ that is expressed in terms of the multiscale geometry of the metric space $(T,\rho )$. A consequence of this result, Theorem 9.7, states that if the metric space has Minkowski dimension $d \ge 1$, then, ignoring the dependence on $\varepsilon$ and lower-order terms in the exponent, we have

1.2 $$\begin{aligned} {{\,\mathrm{{\mathbb {E}}}\,}}W_1 \left( \mu _X,\mu _Y \right) \sim n^{-1/d} \end{aligned}$$

The dependence on *n* is optimal and quite intuitive. Indeed, if the true data *X* consists of *n* i.i.d. random points chosen uniformly from the unit cube $T=[0,1]^d$, then the average spacing between these points is of the order $n^{-1/d}$. So our result shows that privacy can be achieved by a *microscopic perturbation*, one whose magnitude is roughly the same as the average spacing between the points.

Our more general result, Theorem 7.2, holds for arbitrary compact metric spaces $(T,\rho )$ and, more importantly, for general input measures (not just empirical ones). To be able to work in such generality, we employ the notion of *metric privacy* which reduces to differential privacy when we specialize to empirical measures (Sect. 2.1).

### Uniform accuracy over Lipschitz statistics

The choice of the Wasserstein 1-metric to quantify accuracy ensures that all Lipschitz statistics are preserved uniformly. Indeed, by the Kantorovich–Rubinstein duality theorem, (1.1) yields

1.3 $$\begin{aligned} {{\,\mathrm{{\mathbb {E}}}\,}}\sup _f \left| \frac{1}{n} \sum _{i=1}^n f(X_i) - \frac{1}{m} \sum _{i=1}^m f(Y_i) \right| \le \gamma \end{aligned}$$

where the supremum is over all 1-Lipschitz functions $f:T \rightarrow {\mathbb {R}}$.

Standard private synthetic data generation methods that come with rigorous accuracy guarantees do so with respect to a *predefined* set of linear queries, such as low-dimensional marginals, see e.g. [7, 12, 21, 43]. While this may suffice in some cases, there is no assurance that the synthetic data behave in the same way as the original data under more complex, but frequently employed, machine learning techniques. For instance, if we want to apply a clustering method to the synthetic data, we cannot be sure that the results we get are close to those for the true data. This can drastically limit effective and reliable analysis of synthetic data.

In contrast, since the synthetic data constructed via our proposed method satisfy a *uniform* bound (1.3), this provides data analysts with a vastly increased toolbox of machine learning methods for which one can expect outcomes that are similar for the original data and the synthetic data.

As concrete examples let us look at two of the most common tasks in machine learning, namely clustering and classification. While not every clustering method will satisfy a Lipschitz property, there do exist Lipschitz clustering functions that achieve state-of-the-art results, see e.g. [31, 56]. Similarly, there is distinct interest in Lipschitz function based classifiers, since they are more robust and less susceptible to adversarial attacks. This includes conventional classification methods such as support vector machines [51] as well as classifiers based on Lipschitz neural networks [9, 50]. These are just a few examples of complex machine learning tools that can be reliably applied to the synthetic data constructed via our private measure algorithm. Moreover, since our results hold for general compact metric spaces, this paves the way for creating private synthetic data for a wide range of data types. We will present a detailed algorithmic and numerical investigation of the proposed method in a forthcoming paper.

### A superregular random walk

The most popular way to achieve privacy is by adding random noise, typically either by adding an appropriate amount of Laplacian noise or Gaussian noise (these methods are aptly referred to as *Laplacian mechanism* and *Gaussian mechanism*, respectively [22]). We, too, can try to make a probability measure $\mu$ on *T* private by discretizing *T* (replacing it with a finite set of points) and then adding random noise to the weights of the points. Going this route, however, yields suboptimal results. For example, it is not difficult to check that if *T* is the interval [0, 1], the accuracy of the Laplacian mechanism cannot be better than $n^{-1/2}$, which is suboptimal compared to optimal accuracy $n^{-1}$ in (1.2).

This loss of accuracy is caused by the accumulation of additive noise. Indeed, adding *n* independent random variables of unit variance produces noise of the order $n^{1/2}$. This prompts a basic probabilistic question: can we construct *n* random variables that are “close” to being independent, but whose partial sums cancel more perfectly than those of independent random variables? We answer this question affirmatively in Theorem 3.1, where we construct random variables $Z_1,\ldots ,Z_n$ whose joint distribution is as regular as that of i.i.d. Laplacian random variables, yet whose partial sums grow *logarithmically* as opposed to $n^{1/2}$:

$$\begin{aligned} \max _{1 \le k \le n} {{\,\mathrm{{\mathbb {E}}}\,}}\big ( Z_1+\cdots +Z_k \big )^2 = O(\log ^3 n). \end{aligned}$$

One can think of this as a random walk that is locally similar to the one with i.i.d. steps, but is globally much more bounded. Our construction is a nontrivial modification of Lévy’s construction of Brownian motion. It may be interesting and useful beyond applications to privacy.

### Comparison to existing work

The numerically efficient construction of accurate differentially private synthetic data is highly non-trivial. As case in point, Ullman and Vadhan [44] showed (under standard cryptographic assumptions) that in general it is NP-hard to make private synthetic Boolean data which approximately preserve all two-dimensional marginals. There exists a substantial body of work for generating privacy-preserving synthetic data, cf. e.g. [1, 4, 14, 16, 35], but—unlike our work—without providing any rigorous privacy or accuracy guarantees. Those papers on synthetic data that do provide rigorous guarantees are limited to accuracy bounds for a finite set of a priori specified queries, see for example [7, 11–13, 21, 43], see also the tutorial [46]. As discussed before, this may suffice for specific purposes, but in general severely limits the impact and usefulness of synthetic data. In contrast, the present work provides accuracy guarantees for a wide range of machine learning techniques. Furthermore, our our results hold for general compact metric spaces, as we establish metric privacy instead of just differential privacy.

A special example of the topic investigated in this paper is the publication of differentially private histograms, which is a well studied problem in the privacy literature, see e.g. [2, 26, 36, 37, 39, 54, 55, 57] and Chapter 4 in [33]. In the specific context of histograms, the Haar function based approach to construct a superregular random walk proposed in our paper is related to the wavelet-based method [54] and to other hierarchical histogram partitioning methods [26, 39, 57]. Like our approach, [26, 54] obtain consistency of counting queries across the hierarchical levels, owing to the specific way that noise is added. Also, the accuracy bounds obtained in [26, 54] are similar to ours, as they are also polylogarithmic (although we are able to obtain a smaller exponent). There are, however, several *key differences*. While our approach gives a convenient way to generate accurate and differentially private *synthetic data* *Y* from true data *X*, the methods of the aforementioned papers are not suited to create synthetic data. Instead, these methods release answers to queries. Moreover, accuracy is proven for just *a single* given range query and not simultaneously for all queries like we do. This limitation makes it impossible to create accurate synthetic data with the algorithms in [26, 54]. Moreover, unlike the aforementioned papers, our work allows the data to be quite general, since we prove metric privacy and not just differential privacy. Furthermore, our results apply to multi-dimensional data, and are not limited to the one-dimensional setting.

Perhaps closest to our paper is [52], where the authors consider differentially private synthetic data in $[0, 1]^d$ with guarantees for any smooth queries with bounded partial derivatives of order *K*. The case $K=1$ corresponds to 1-Lipschitz functions considered in our paper. In that case [52] obtains an accuracy of ${{{\mathcal {O}}}}( n^{-\frac{1}{2d+1}})$, while we achieve an accuracy of $\tilde{{{{\mathcal {O}}}}}(n^{-\frac{1}{d}})$.

There exist several papers on the private estimation of density and other statistical quantities [18, 27], and sampling from distributions in a private manner is the topic of [40]. While definitely interesting, that line of work is not concerned with synthetic data, and thus there is little overlap with this work.

### The architecture of the paper

The remainder of this paper is organized as follows. We introduce some background material and notation in Sect. 2, such as the concept of *metric privacy* which generalizes differential privacy. In Sect. 3 we construct a *superregular random walk* (Theorem 3.1). We analyze metric privacy in more detail in Sect. 4, where we also provide a link from the general private measure problem to private synthetic data (Lemma 4.1). In Sect. 5 we use the superregular random walk to construct a *private measure on the interval* [0, 1] (Theorem 5.4). In Sect. 6 we use a link between the Traveling Salesman Problem and minimum spanning trees to devise a *folding technique*, which we apply in Sect. 7 to “fold” the interval into a space-filling curve to construct a *private measure on a general metric space* (Theorem 7.2). Postprocessing the private measure with quantization and splitting, we then generate *private synthetic data* in a general metric space (Corollary 7.4). In Sect. 8 we turn to *lower bounds* for private measures (Theorem 8.5) and synthetic data (Theorem 8.6) on a general metric space. We do this by employing a technique of Hardt and Talwar, which we present in a Proposition 8.1 that identifies general limitations for synthetic data. In Sect. 9 we illustrate our general results on a specific example of a metric space: the Boolean cube $[0,1]^d$. We construct a private measure (Corollary 9.1) and private synthetic data (Corollary 9.2) on the cube, and show near optimality of these results in Corollary 9.3 and Corollary 9.4, respectively. Results similar to the ones for the *d*-dimensional cube hold for arbitrary metric space of Minkowski dimension *d*. For any such space, we prove *asymptotically sharp min-max results* for private measures (Theorem 9.6) and synthetic data (Theorem 9.7).

## Background and notation

The motivation behind the concept of differential privacy is the desire to protect an individual’s data, while publishing aggregate information about the database [22]. Adding or removing the data of one individual should have a negligible effect on the query outcome, as formalized in the following definition.

### Definition 2.1

(*Differential Privacy* [22]) A randomized algorithm ${{\mathcal {M}}}$ gives $\varepsilon$-differential privacy if for any input databases *D* and $D'$ differing on at most one element, and any measurable subset $S \subseteq {{\,\textrm{range}\,}}({{\mathcal {M}}})$, we havewhere the probability is with respect to the randomness of ${{\mathcal {M}}}$.

### Defining metric privacy

While differential privacy is a concept of the discrete world (where datasets can differ in a *single element*), it is often desirable to have more freedom in the choice of input data. The following general notion (which seems to be known under slightly different, and somewhat less general, versions, see e.g. [5] and the references therein) extends the classical concept of differential privacy.

#### Definition 2.2

(*Metric privacy*) Let $(T,\rho )$ be a compact metric space and *E* be a measurable space. A randomized algorithm ${\mathcal {A}}: T \rightarrow E$ is called $\alpha$-metrically private if, for any inputs $x,x' \in T$ and any measurable subset $S \subset E$, we have2.1

To see how this metric privacy encompasses differential privacy, consider a product space $T = {\mathcal {X}}^n$ and equip it with the Hamming distance

2.2 $$\begin{aligned} \rho _H(x,x') = \left| {\{i \in [n]:\; x_i \ne x'_i\}}\right| . \end{aligned}$$

The $\alpha$-differentially privacy of an algorithm ${\mathcal {A}}: {\mathcal {X}}^n \rightarrow E$ can be expressed as2.3Note that (2.3) is equivalent to (2.1) for $\rho =\rho _H$. Obviously, (2.1) implies (2.3). The converse implication can be proved by replacing one coordinate of *x* by the corresponding coordinate of $x'$ and applying (2.3) $\rho _H(x,x')$ times, then telescoping. Let us summarize:

#### Lemma 2.3

(MP vs. DP) Let ${\mathcal {X}}$ be an arbitrary set. Then an algorithm ${\mathcal {A}}: {\mathcal {X}}^n \rightarrow E$ is $\alpha$-differentially private if an only if ${\mathcal {A}}$ is $\alpha$-metrically private with respect to the Hamming distance (2.2) on ${\mathcal {X}}^n$.

Unlike differential privacy, metric privacy goes beyond product spaces, and thus allows the data to be quite general. In this paper, for example, the input data are probability measures. Moreover, metric privacy does away with the assumption that the data sets $D, D'$ be different in a *single element*. This assumption is sometimes too restrictive: general measures, for example, do not break down into natural single elements.

### Distances between measures

This paper will use three classical notions of distance between measures.

#### Total variation

The total variation (TV) norm [19, Section III.1] of a signed measure $\mu$ on a measurable space $(\Omega ,{\mathcal {F}})$ is defined as^1^

2.4 $$\begin{aligned} \Vert {\mu } \Vert _\textrm{TV}= \frac{1}{2} \sup _{\Omega = \cup _i A_i} \sum _i \left| {\mu (A_i)}\right| \end{aligned}$$

where the supremum is over all partitions $\Omega$ into countably many parts $A_i \in {\mathcal {F}}$. If $\Omega$ is countable, we have

2.5 $$\begin{aligned} \Vert {\mu } \Vert _\textrm{TV}= \frac{1}{2} \sum _{\omega \in \Omega } \left| {\mu (\{\omega \})}\right| . \end{aligned}$$

The TV distance between two probability measures $\mu$ and $\nu$ is defined as the TV norm of the signed measure $\mu -\nu$. Equivalently,

$$\begin{aligned} \Vert {\mu -\nu } \Vert _\textrm{TV}= \sup _{A \in {\mathcal {F}}} \left| {\mu (A)-\nu (A)}\right| . \end{aligned}$$

#### Wasserstein distance

Let $(\Omega ,\rho )$ be a bounded metric space. We define the Wasserstein 1-distance (henceforth simply referred to as Wasserstein distance) between probability measures $\mu$ and $\nu$ on $\Omega$ as [49]

2.6 $$\begin{aligned} W_1(\mu ,\nu ) = \inf _\gamma \int _{\Omega \times \Omega } \rho (x,y) \, d\gamma (x,y) \end{aligned}$$

where the infimum is over all *couplings* $\gamma$ of $\mu$ and $\nu$, or probability measures on $\Omega \times \Omega$ whose marginals on the first and second coordinates are $\mu$ and $\nu$, respectively. In other words, $W_1(\nu ,\mu )$ minimizes the *transportation cost* between the “piles of earth” $\mu$ and $\nu$.

The Kantorovich–Rubinstein duality theorem [49] gives an equivalent representation:

$$\begin{aligned} W_1(\mu ,\nu ) = \sup _{\Vert {h} \Vert _\textrm{Lip}\le 1} \left( \int h\, d\mu - \int h\, d\nu \right) \end{aligned}$$

where the supremum is over all continuous, 1-Lipschitz functions $h: \Omega \rightarrow {\mathbb {R}}$.

For probability measures $\mu$ and $\nu$ on ${\mathbb {R}}$, the Wasserstein distance has the following representation, according to Vallender [47]:

2.7 $$\begin{aligned} W_1(\mu ,\nu ) = \Vert {F_\mu -F_\nu } \Vert _{L^1({\mathbb {R}})}. \end{aligned}$$

Here $F_\mu (x) = \mu \left( (-\infty ,x] \right)$ is the cumulative distribution function of $\mu$, and similarly for $F_\nu (x)$.

Vallender’s identity (2.7) can be used to define Wasserstein distance for *signed measures* on ${\mathbb {R}}$. Moreover, for signed measures on [0, 1], the Wasserstein distance defined this way is always finite, and it defines a pseudometric.

## A superregular random walk

The classical random walk with independent steps of unit variance is not bounded: it deviates from the origin at the expected rate $\sim n^{1/2}$. Surprisingly, there exists a random walk whose joint distribution of steps is as regular as that of independent Laplacians, yet that deviates from the origin *logarithmically* slowly.

### Theorem 3.1

(A superregular random walk) For every $n \in {\mathbb {N}}$, there exists a probability density of the form $f(z) = \frac{1}{\beta } e^{-V(z)}$ on ${\mathbb {R}}^n$ that satisfies the following two properties.

(i) (Regularity): the potential *V* is 1-Lipschitz in the $\ell ^1$ norm, i.e.
  3.1 $$\begin{aligned} \left| {V(x)-V(y)}\right| \le \Vert {x-y} \Vert _1 \quad \text {for all } x,y \in {\mathbb {R}}^n. \end{aligned}$$
(ii) (Boundedness): a random vector $Z=(Z_1,\ldots ,Z_n)$ distributed according to the density *f* satisfies
  3.2 $$\begin{aligned} {{\,\mathrm{{\mathbb {E}}}\,}}(Z_1+\cdots +Z_k)^2 \le C \log ^3 n \quad \text {for all } 1 \le k \le n, \end{aligned}$$
  where $C>0$ is a universal constant.

### Heuristics

We will define a superregular random walk by modifying the Lévy’s construction of the Brownian motion. In this construction, the path of a Brownian motion on [0, 1] is defined as a random Gaussian series with respect to the Faber–Schauder basis of the space of continuous functions, see [10, Section IX.1]. We will replace Gaussian weights by Laplacian weights with smaller variances, and truncate the series.

To that end, recall the definition of the *Faber–Schauder system* of “hat functions” $\phi _1,\phi _2,\ldots$ on the interval [0, 1]. First, we set

$$\begin{aligned} \phi _1(t) = t. \end{aligned}$$

Next, for each level $\ell \in {\mathbb {N}}$ and each $k \in \{1,\ldots ,2^{\ell -1}\}$, we define $\phi _{2^{\ell -1}+k}(t)$ as the function on [0, 1] that takes value 0 outside the interval

3.3 $$\begin{aligned} (a_{\ell ,k}, b_{\ell ,k}) :=\left( \frac{k-1}{2^{\ell -1}}, \, \frac{k}{2^{\ell -1}} \right) , \end{aligned}$$

takes value 1 at the midpoint $c_k :=(2k-1)/2^\ell$ of the interval, and interpolates linearly in between. The Faber–Schauder system forms a Shcauder basis in the Banach space $C_0[0,1]$ of continuous functions that take zero value at the origin.

**Fig. 1:**
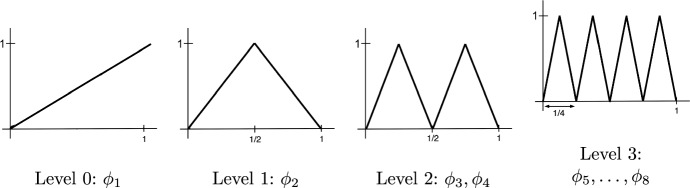
Faber–Schauder system

The Faber–Schauder system is conveniently organized by levels $\ell =0,1,2,\ldots$ We place a single function $\phi _1(t)=t$ is on level $\ell =0$, and each subsequent level $\ell \ge 1$ contains $2^{\ell -1}$ functions $\phi _j$ supported on disjoint intervals $(a_{\ell ,k}, b_{\ell ,k})$ of length $1/2^{\ell -1}$. Throughout this section, $\ell (j)$ will denote the level the function $\phi _j$ belongs to, e.g. $\ell (1)=0$, $\ell (2)=1$, $\ell (3)=\ell (4)=2$, $\ell (5)=\ell (6)=\ell (7)=\ell (8)=3$, etc. See Fig. 1 for an illustration of these functions.

Lévy’s definition of the standard Brownian motion on the interval [0, 1] is

3.4 $$\begin{aligned} B_n(t) = \sum _{j=1}^\infty G_j \phi _j(t), \end{aligned}$$

where $G_j$ are independent normal random variables, namely

$$\begin{aligned} G_1 \sim N(0,1); \quad G_j \sim N \big ( 0, 2^{-\ell (j)-1} \big ), \, j=2,3,\ldots . \end{aligned}$$

To construct a superregular random walk, we replace the Gaussian weights $G_j$ by Laplacian^2^ weights $\Lambda _j \sim {{\,\textrm{Lap}\,}}(\log n)$. and we truncate the series at *n*. Thus, we set

3.5 $$\begin{aligned} W_n(t) = \sum _{j=1}^n \Lambda _j \phi _j(t). \end{aligned}$$

The superregular random walk could then be defined as

3.6 $$\begin{aligned} Z_1+\cdots +Z_k = W_n(k/n), \quad k=1,\ldots ,n. \end{aligned}$$

### Formal construction

First observe that the regularity property (3.1) of a probability distribution on ${\mathbb {R}}^{n}$ passes on to the marginal distributions. For example, regularity of a random vector $(X_1,X_2) \in {\mathbb {R}}^2$ means that

$$\begin{aligned} f_{(X_1,X_2)}(x_1,x_2) \le \exp (-\left| {x_1-y_1}\right| -\left| {x_2-y_2}\right| ) \, f_{(X_1,X_2)}(y_1,y_2), \end{aligned}$$

for all $(x_1,y_1),(x_2,y_2)\in {\mathbb {R}}^{2}$. In particular,

$$\begin{aligned} f_{(X_1,X_2)}(x_1,x_2) \le \exp (-\left| {x_2-y_2}\right| ) \, f_{(X_1,X_2)}(x_1,y_2). \end{aligned}$$

Taking integral with respect to $x_1$ on both sides yields

$$\begin{aligned} f_{X_2}(x_2) \le \exp (-\left| {x_2-y_2}\right| ) \, f_{X_2}(y_2), \end{aligned}$$

which is equivalent to the regularity of the random vector $X_2 \in {\mathbb {R}}^1$. The same argument works in higher dimensions.

Thus, by dropping at most *n*/2 terms if necessary, we can assume without loss of generality that

3.7 $$\begin{aligned} n=2^L \quad \text {for some } L \in {\mathbb {N}}, \end{aligned}$$

Thus, the Faber–Schauder functions $\phi _1,\ldots ,\phi _n$ are partitioned in $L+1$ full levels $0,1,\ldots ,L$. Consider i.i.d. random variables

3.8 $$\begin{aligned} \Lambda _1,\ldots ,\Lambda _n \sim {{\,\textrm{Lap}\,}}(2L+1), \end{aligned}$$

define the random process $W_n(t)$ by Eq. (3.5), and define the random variables $Z_1,\ldots ,Z_k$ by Eq. (3.6).

The construction is complete. It remains to check boundedness and regularity.

### Boundedness

Fix $k \in [n]$. We have

3.9 $$\begin{aligned} {{\,\mathrm{{\mathbb {E}}}\,}}(Z_1+\cdots +Z_k)^2&= {{\,\mathrm{{\mathbb {E}}}\,}}\left( \sum _{j=1}^n \Lambda _j \, \phi _j(k/n) \right) ^2 \quad \text {(by definition)} \nonumber \\&= \sum _{j=1}^n {{\,\mathrm{{\mathbb {E}}}\,}}[\Lambda _j^2] \, \phi _j(k/n)^2 \quad \text {(by independence and mean zero)} \nonumber \\&= 2(2L+1)^2 \sum _{j=1}^n \phi _j(k/n)^2 \quad \text {(by }\text {(}3.8\text {))}. \end{aligned}$$

By construction, the Faber–Schauder functions $\phi _j$ on each given level have disjoint support. Thus, on each level there can be be at most one function that makes the value $\phi _j(k/n)^2$ nonzero. By construction, this value is bounded by 1. Adding these values for the $L+1$ levels, we conclude that $\sum _{j=1}^n \phi _j(k/n)^2$ is bounded by $L+1$. Hence

$$\begin{aligned} {{\,\mathrm{{\mathbb {E}}}\,}}(Z_1+\cdots +Z_k)^2 \le 2(2L+1)^2 (L+1) \lesssim \log ^3 n \end{aligned}$$

where we used (3.7) in the last step.

### Regularity

By definition (3.5) and (3.6), we have

$$\begin{aligned} Z_k = W_n \left( \frac{k}{n} \right) - W_n \left( \frac{k-1}{n} \right) = \sum _{j=1}^n \Lambda _j \psi _j(k) \end{aligned}$$

where

$$\begin{aligned} \psi _j(k) = \phi _j \left( \frac{k}{n} \right) - \phi _j \left( \frac{k-1}{n} \right) , \quad k=1,\ldots ,n. \end{aligned}$$

**Fig. 2:**
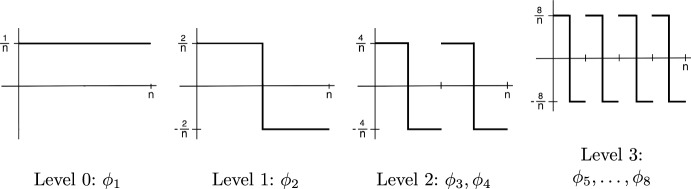
Haar system

The discrete functions $\psi _j$ can be thought as (discrete) derivatives of the Faber–Schauder functions $\phi _j$, and they are known as (discrete, rescaled) *Haar system*, cf. [10, 41]. The Haar basis is illustrated in Fig. 2.

The Haar system $\psi _1,\ldots ,\psi _n$ form an orthogonal basis of $\ell ^2[n]$, see [10]. Thus, every function $x \in \ell ^2[n]$ admits the orthogonal decomposition

$$\begin{aligned} x = \sum _{j=1}^n \lambda (x)_j \, \psi _j \quad \text {where } \lambda (x)_j = \frac{\langle \psi _j,x\rangle }{\Vert {\psi _j} \Vert _2^2}, \end{aligned}$$

where $\langle ,\rangle$ and $\Vert \,\Vert _2$ are the standard inner product and norm on $\ell ^{2}[n]$.

The key property of the coefficient vector $\lambda (x)$ is its approximate sparsity, which we can express via the $\ell ^1$ norm.

#### Lemma 3.2

(Sparsity) For any function $x \in \ell ^2[n]$, the coefficient vector $\lambda (x)$ satisfies

$$\begin{aligned} \Vert {\lambda (x)} \Vert _1 \le (2L+1) \Vert {x} \Vert _1. \end{aligned}$$

#### Proof

First, let us prove the lemma for the indicator of any single point $k \in [n]$, i.e. for $x={{{\textbf {1}}}}_{\{k\}}$. Here we have

$$\begin{aligned} \lambda (x)_j = \frac{\psi _j(k)}{\Vert {\psi _j} \Vert _2^2}. \end{aligned}$$

First, consider $j=1$, the only index on level $\ell =0$. The function $\psi _1(t)=1/n$ trivially satisfies $\psi _1(k)=1/n$ and $\Vert {\psi _j} \Vert _2^2 = 1/n$, so we have $\lambda (x)_1=1$.

Next, consider an index *j* on some level $\ell \ge 1$. By construction, any function $\psi _j$ on that level can takes on three values: 0 and $\pm 2^\ell /n$. Moreover, $\psi _j$ is supported on an interval of length $n/2^{\ell -1}$, see (3.3). Hence $\Vert {\psi _j} \Vert _2^2 = 2^{\ell +1}/n$, so $\left| {\lambda (x)_j}\right| \le 2$.

Moreover, the functions $\psi _j$ on any given level have disjoint support. So among all such functions on each level, at most one can make the value $\psi _j(k)$ and thus $\lambda (x)_j$ nonzero. As we just showed, for level 0 this value is 1, and for each subsequent level $\ell \in \{1,\ldots ,L\}$, this value is bounded by 2. Summing over all levels, we conclude that $\Vert {\lambda (x)} \Vert _1 \le 2\,L+1$.

To extend this bound to a general function $x \in \ell ^2[n]$, decompose it as $x = \sum _{k=1}^n x(k) {{{\textbf {1}}}}_{\{k\}}$. Then, by linearity, $\lambda (x) = \sum _{k=1}^n x(k) \lambda ({{{\textbf {1}}}}_{\{k\}})$, so

$$\begin{aligned} \Vert {\lambda (x)} \Vert _1 \le \sum _{k=1}^n \left| {x(k)}\right| \, \Vert {\lambda ({{{\textbf {1}}}}_{\{k\}})} \Vert _1. \end{aligned}$$

The bound $\Vert {\lambda ({{{\textbf {1}}}}_{\{k\}})} \Vert _1 \le 2L+1$ from the first part of the argument completes the proof of the lemma. $\square$

We are ready to prove regularity. Consider the random function $Z = \sum _{j=1}^n \Lambda _j \psi _j$ constructed in Sect. 3.2. In our new notation, the coefficient vector of *Z* is $\lambda (Z)=(\Lambda _1,\ldots ,\Lambda _n)=:\Lambda$. We have for any $x,y \in \ell ^2[n]$:

3.10 $$\begin{aligned} r(x,y) :=\frac{{{\,\textrm{dens}\,}}_X(x)}{{{\,\textrm{dens}\,}}_X(y)} = \frac{{{\,\textrm{dens}\,}}_\Lambda (\lambda (x))}{{{\,\textrm{dens}\,}}_\Lambda (\lambda (y))}. \end{aligned}$$

To see this, recall that the map $x \mapsto \lambda (x)$ is a linear bijection on $\ell ^2[n]$. Hence for any $\varepsilon > 0$ and for the unit ball *B* of $\ell ^2[n]$, we haveTaking the limit on both sides as $\varepsilon \rightarrow 0_+$ and applying the Lebesgue differentiation theorem yield (3.10).

By construction, the coefficients $\Lambda _i$ of the random vector $\Lambda \in {\mathbb {R}}^n$ are ${{\,\textrm{Lap}\,}}(2L+1)$ i.i.d. random variables. Hence

$$\begin{aligned} {{\,\textrm{dens}\,}}_\Lambda (z) = \frac{1}{\big ( 2(2L+1) \big )^n} \exp \Big ( -\frac{\Vert {z} \Vert _1}{2L+1} \Big ), \quad z \in {\mathbb {R}}^n. \end{aligned}$$

Thus,

$$\begin{aligned} r(x,y) = \exp \Bigg ( \frac{\Vert {\lambda (y)} \Vert _1 - \Vert {\lambda (x)} \Vert _1}{2L+1} \Bigg ). \end{aligned}$$

By the triangle inequality and Lemma 3.2, we have

$$\begin{aligned} \Vert {\lambda (y)} \Vert _1 - \Vert {\lambda (x)} \Vert _1 \le \Vert {\lambda (x)-\lambda (y)} \Vert _1 \le (2L+1) \Vert {x-y} \Vert _1. \end{aligned}$$

Thus

$$\begin{aligned} r(x,y) \le \exp (\Vert {x-y} \Vert _1). \end{aligned}$$

If we express the density in the form ${{\,\textrm{dens}\,}}_X(x) = \frac{1}{\beta } e^{-V}$, the bound we proved can be written as

$$\begin{aligned} \exp \left( V(y)-V(x) \right) \le \exp (\Vert {x-y} \Vert _1), \end{aligned}$$

or $V(y)-V(x) \le \Vert {x-y} \Vert _1$. Swapping *x* with *y* yields $\left| {V(x)-V(y)}\right| \le \Vert {x-y} \Vert _1$. The proof of Theorem 3.1 is complete. $\square$

#### Remark 3.3

(Boundedness of paths) One can easily upgrade the bound (3.2), which holds in expectation, into a concentration bound that holds with high probability. To do so, instead of applying the additivity of variance in (3.9), one can use a concentration inequality for sums of independent random variables, e.g. Bernstein’s. Moreover, combining the resulting high-probability bound with a union bound, one can obtain boundedness of the entire paths of the random walk, showing that

$$\begin{aligned} {{\,\mathrm{{\mathbb {E}}}\,}}\max _{1 \le k \le n} \big ( Z_1+\cdots +Z_k \big )^2 \le C \log ^4 n. \end{aligned}$$

Since we do not need this result for our application, we leave it to the interested reader.

### Beyond the $\ell ^1$ norm?

One may wonder why specifically the $\ell ^1$ norm appears in the regularity property of Theorem 3.1. As we will see shortly, the regularity with respect to the $\ell ^1$ norm is exactly what is needed in our applications to privacy. However, it might be interesting to see if there are natural extensions of Theorem 3.1 for general $\ell ^p$ norms. The lemma below rules out one such avenue, showing that if a potential *V* is Lipschitz with respect to the $\ell ^p$ norm for some $p>1$, the corresponding random walk deviates at least *polynomially* fast (as opposed to logarithmically fast).

#### Proposition 3.4

(No boundedness for $\ell ^p$-regular potentials) Let $n \in {\mathbb {N}}$ and consider a probability density of the form $f(z)=\frac{1}{\beta }e^{-V(z)}$ on ${\mathbb {R}}^{n}$. Assume that the potential *V* is 1-Lipschitz in the $\ell ^p$-norm. Then a random vector $Z = (Z_1,\ldots ,Z_n)$ distributed according to the density *f* satisfies

$$\begin{aligned} {{\,\mathrm{{\mathbb {E}}}\,}}\left| {Z_1+\cdots +Z_n}\right| \ge \frac{1}{4} n^{1-\frac{1}{p}}. \end{aligned}$$

#### Proof

We can write $Z_1+\cdots +Z_n = \langle Z,u\rangle$ where $u = (1,\ldots ,1)^{\textsf{T}}$. Since $\Vert {n^{-\frac{1}{p}}u} \Vert _p=1$ and *V* is 1-Lipschitz in the $\ell ^{p}$ norm, the densities of the random vectors $Z+n^{-\frac{1}{p}}u$ and *Z* differ by a multiplicative factor of at most *e* pointwise. Therefore,

$$\begin{aligned} {{\,\mathrm{{\mathbb {E}}}\,}}\left| {\langle Z,u\rangle }\right|&\ge e^{-1} {{\,\mathrm{{\mathbb {E}}}\,}}\left| {\langle Z+n^{-\frac{1}{p}}u,u\rangle }\right| \\&\ge e^{-1} \Big ( \left| {\langle n^{-\frac{1}{p}}u,u\rangle }\right| - {{\,\mathrm{{\mathbb {E}}}\,}}\left| {\langle Z,u\rangle }\right| \Big ) \quad \text {(by triangle inequality)} \\&= e^{-1} \Big ( n^{1-\frac{1}{p}} - {{\,\mathrm{{\mathbb {E}}}\,}}\left| {\langle Z,u\rangle }\right| \Big ). \end{aligned}$$

Rearranging the terms, we deduce that

$$\begin{aligned} {{\,\mathrm{{\mathbb {E}}}\,}}\left| {\langle Z,u\rangle }\right| \ge \frac{e^{-1}}{1+e^{-1}} n^{1-\frac{1}{p}} \ge \frac{1}{4} n^{1-\frac{1}{p}}, \end{aligned}$$

which completes the proof. $\square$

In light of Theorem 3.1 and Proposition 3.4 it might be interesting to see if an obstacle remains for the density $f(z)=\frac{1}{\beta }e^{-V(z)^p}$ for $p>1$.

## Metric privacy

### Private measures

The superregular random walk we just constructed will become the main tool in solving the following *private measure problem*. We are looking for a private and accurate algorithm ${\mathcal {A}}$ that transforms a probability measure $\mu$ on a metric space $(T,\rho )$ into another finitely-supported probability measure ${\mathcal {A}}(\mu )$ on $(T,\rho )$.

We need to specify what we mean by privacy and accuracy here. *Metric privacy* offers a natural framework for our problem. Namely, we consider Definition 2.2 for the space $({\mathcal {M}}(T), \textrm{TV})$ of all probability measures on *T* equipped with the TV metric (recalled in Sect. 2.2.1). Thus, for any pair of input measures $\mu$ and $\mu '$ on *T* that are close in the TV metric, we would like the distributions of the (random) output measures ${\mathcal {A}}(\mu )$ and ${\mathcal {A}}(\mu ')$ to be close:4.1The accuracy will be measured via the *Wasserstein distance* (recalled in Sect. 2.2.2). We hope to make $W_1({\mathcal {A}}(\mu ),\mu )$ as small as possible. The reason for choosing $W_1$ as distance is that it allows us to derive accuracy guarantees for general Lipschitz statistics, as outlined below.

### Synthetic data

The private measure problem has an immediate application for *differentially private synthetic data*. Let $(T,\rho )$ be a compact metric space. We hope to find an algorithm ${\mathcal {B}}$ that transforms the true data $X =(X_1,\ldots ,X_n) \in T^n$ into synthetic data $Y=(Y_1,\ldots ,Y_m) \in T^m$ for some *m* such that the empirical measures

$$\begin{aligned} \mu _X = \frac{1}{n} \sum _{i=1}^n \delta _{X_i} \quad \text {and} \quad \mu _Y = \frac{1}{m} \sum _{i=1}^m \delta _{Y_i} \end{aligned}$$

are close in the Wasserstein distance, i.e. we hope to make $W_1(\mu _X,\mu _Y)$ small. This would imply that synthetic data accurately preserves all Lipschitz statistics, i.e.

$$\begin{aligned} \frac{1}{n} \sum _{i=1}^n f(X_i) \approx \frac{1}{m} \sum _{i=1}^m f(Y_i) \end{aligned}$$

for any Lipschitz function $f:T \rightarrow {\mathbb {R}}$.

This goal can be immediately achieved if we solve a version of the private measure problem, described in Sect. 4.1, with the additional requirement that ${\mathcal {A}}(\mu )$ be an empirical measure. Indeed, define the algorithm ${\mathcal {B}}$ by feeding the empirical measure $\mu _X$ into ${\mathcal {A}}$, i.e. set ${\mathcal {B}}(X) = {\mathcal {A}}(\mu _X)$. The accuracy follows, and the differential privacy of ${\mathcal {B}}$ can be seen as follows.

For any pair $X,X'$ of input data that differ in a single element, the corresponding empirical measures differ by at most 1/*n* with respect to the TV distance, i.e.

$$\begin{aligned} \Vert {\mu _X-\mu _{X'}} \Vert _\textrm{TV}\le \frac{1}{n}. \end{aligned}$$

Then, for any subset *S* in the output space, we can use (4.1) to getThus, if $\alpha = \varepsilon n$, the algorithm ${\mathcal {B}}$ is $\varepsilon$-differentially private. Let us record this observation formally.

#### Lemma 4.1

(Private measure yields private synthetic data) Let $(T,\rho )$ be a compact metric space. Let ${\mathcal {A}}$ be an algorithm that inputs a probability measure on *T*, and outputs something. Define the algorithm ${\mathcal {B}}$ that takes data $X =(X_1,\ldots ,X_n) \in T^n$ as an input, creates the empirical measure $\mu _X$ and feeds it into the algorithm ${\mathcal {A}}$, i.e. set ${\mathcal {B}}(X) = {\mathcal {A}}(\mu _X)$. If ${\mathcal {A}}$ is $\alpha$-metrically private in the TV metric and $\alpha = \varepsilon n$, then ${\mathcal {B}}$ is $\varepsilon$-differentially private.

Thus, our main focus from now on will be on solving the private measure problem; private synthetic data will follows as a consequence.

## A private measure on the line

In this section, we construct a private measure on the interval [0, 1]. Later we will extend this construction to general metric spaces.

### Discrete input space

Let us start with a somewhat restricted goal, and then work toward wider generality. In this subsection, we will (a) assume that the input measure $\mu$ is always supported on some fixed finite subset

$$\begin{aligned} \Omega = \{\omega _1,\ldots ,\omega _n\} \quad \text {where } 0 \le \omega _1 \le \cdots \le \omega _n \le 1 \end{aligned}$$

and (b) allow the output ${\mathcal {A}}(\mu )$ to be a signed measure. We will measure accuracy with the Wasserstein distance.

#### Perturbing a measure by a superregular random walk

Apply the Superregular Random Walk Theorem 3.1 and rescale the random variables $Z_i$ by setting $U_i = (2/\alpha ) Z_i$. The regularity property of the random vector $U = (U_1,\ldots ,U_n)$ takes the form

5.1 $$\begin{aligned} \frac{{{\,\textrm{dens}\,}}_U(x)}{{{\,\textrm{dens}\,}}_U(y)} \le \exp \left( \frac{\alpha }{2} \Vert {x-y} \Vert _1 \right) \quad \text {for all } x,y \in {\mathbb {R}}^n, \end{aligned}$$

and the boundedness property implies that

5.2 $$\begin{aligned} \max _{1 \le k \le n}{{\,\mathrm{{\mathbb {E}}}\,}}\left| {U_1+\cdots +U_k}\right| \le \frac{C \log ^{\frac{3}{2}} n}{\alpha }. \end{aligned}$$

Let us make the algorithm ${\mathcal {A}}$ perturb the measure $\mu$ on $\Omega$ by the weights $U_i$, i.e. we set

5.3 $$\begin{aligned} {\mathcal {A}}(\mu )(\omega _i) = \mu (\{\omega _i\})+U_i, \quad i=1,\ldots ,n. \end{aligned}$$

#### Privacy

Any measure $\nu$ on $\Omega$ can be identified with the vector ${\bar{\nu }} \in {\mathbb {R}}^n$ by setting ${\bar{\nu }}_i = \nu (\{\omega _i\})$. Then, for any measure $\eta$ on $\Omega$, we have

5.4 $$\begin{aligned} {{\,\textrm{dens}\,}}_{{\mathcal {A}}(\mu )}(\eta ) = {{\,\textrm{dens}\,}}_{{\bar{\mu }}+U}({\bar{\eta }}) = {{\,\textrm{dens}\,}}_{U}({\bar{\eta }}-{\bar{\mu }}). \end{aligned}$$

Fix two measures $\mu$ and $\mu '$ on $\Omega$. By above, we have

$$\begin{aligned} \frac{{{\,\textrm{dens}\,}}_{{\mathcal {A}}(\mu )}(\eta )}{{{\,\textrm{dens}\,}}_{{\mathcal {A}}(\mu ')}(\eta )}&= \frac{{{\,\textrm{dens}\,}}_{U}({\bar{\eta }}-{\bar{\mu }})}{{{\,\textrm{dens}\,}}_{U}({\bar{\eta }}-\bar{\mu '})} \quad \text {(by (}5.4\text {))}\\&\le \exp \left( \frac{\alpha }{2} \Vert {{\bar{\mu }}-\bar{\mu '}} \Vert _1 \right) \quad \text {(by (}5.1\text {))}\\&= \exp \left( \alpha \Vert {\mu -\mu '} \Vert _\textrm{TV}\right) \quad \text {(by (}2.5\text {))}. \end{aligned}$$

This shows that the algorithm ${\mathcal {A}}$ is $\alpha$-metrically private in the TV metric.

#### Accuracy

By definition definition (2.7) of Wasserstein distance for signed measures, we have

$$\begin{aligned}&{{\,\mathrm{{\mathbb {E}}}\,}}W_{1}({\mathcal {A}}(\mu ),\mu )\\&\quad = \int _{0}^{1} {{\,\mathrm{{\mathbb {E}}}\,}}\left| {\big ( {\mathcal {A}}(\mu )-\mu \big ) \big ( [0,x] \big )}\right| \,dx \quad \text {(using linearity of expectation)} \\&\quad = \int _{0}^{1} {{\,\mathrm{{\mathbb {E}}}\,}}\left| {\sum _{j: \, \omega _j \le x} \big ( {\mathcal {A}}(\mu )-\mu \big ) (\omega _j)}\right| \,dx \quad \text {(measures are supported on points }\omega _j\text {)} \\&\quad = \int _{0}^{1} {{\,\mathrm{{\mathbb {E}}}\,}}\left| {\sum _{j=1}^{k(x)} U_j}\right| \,dx \quad \text {(by (}5.3\text {), where we set }k(x) = \left| {\{j:\, \omega _j \le x\}}\right| \text {)}\\&\quad \le \max _{1 \le k \le n} {{\,\mathrm{{\mathbb {E}}}\,}}\left| {\sum _{j=1}^k U_j}\right| \le \frac{C \log ^{\frac{3}{2}} n}{\alpha } \quad \text {(by (}5.2\text {))}. \end{aligned}$$

The following result summarizes what we have proved.

##### Proposition 5.1

(Input in discrete space, output signed measure) Let $\Omega$ be finite subset of [0, 1] and let $n = \left| {\Omega }\right|$. Let $\alpha >0$. There exists a randomized algorithm ${\mathcal {A}}$ that takes a probability measure $\mu$ on $\Omega$ as an input and returns a signed measure $\nu$ on $\Omega$ as an output, and with the following two properties.

(i) (Privacy): the algorithm ${\mathcal {A}}$ is $\alpha$-metrically private in the TV metric.
(ii) (Accuracy): for any input measure $\mu$, the expected accuracy of the output signed measure $\nu$ in the Wasserstein distance is
  $$\begin{aligned} {{\,\mathrm{{\mathbb {E}}}\,}}W_{1}(\nu ,\mu ) \le \frac{C \log ^{\frac{3}{2}} n}{\alpha }. \end{aligned}$$

Let $\nu$ be the signed measure obtained in Proposition 5.1. Let ${\widehat{\nu }}$ be a probability measure on $\Omega$ that minimizes $W_{1}({\widehat{\nu }},\nu )$. In view of (2.7), finding ${\widehat{\nu }}$ can be cast as convex problem, although the minimizer may not be unique. By minimality, $W_{1}({\widehat{\nu }},\nu )\le W_{1}(\mu ,\nu )$. So $W_{1}({\widehat{\nu }},\mu )\le W_{1}({\widehat{\nu }},\nu )+W_{1}(\nu ,\mu )\le 2W_{1}(\mu ,\nu )$. Thus, we can upgrade the previous result, making the output a measure (as opposed to signed measure):

##### Proposition 5.2

(Private measure on a finite subset of the interval) Let $\Omega$ be finite subset of [0, 1] and let $n = \left| {\Omega }\right|$. Let $\alpha >0$. There exists a randomized algorithm ${\mathcal {B}}$ that takes a probability measure $\mu$ on $\Omega$ as an input and returns a probability measure $\nu$ on $\Omega$ as an output, and with the following two properties.

(i) (Privacy): the algorithm ${\mathcal {B}}$ is $\alpha$-metrically private in the TV metric.
(ii) (Accuracy): for any input measure $\mu$, the expected accuracy of the output measure $\nu$ in the Wasserstein distance is
  $$\begin{aligned} {{\,\mathrm{{\mathbb {E}}}\,}}W_{1}(\nu ,\mu ) \le \frac{C \log ^{\frac{3}{2}} n}{\alpha }. \end{aligned}$$

### Extending the input space to the interval

Next, we would like to extend our framework to a continuous setting, and allow measures to be supported by the entire interval [0, 1]. We can do this by quantization.

#### Quantization

Fix $n \in {\mathcal {N}}$ and let ${\mathcal {N}}= \{\omega _1,\ldots ,\omega _n\}$ be a (1/*n*)-net of [0, 1]. Consider the proximity partition

$$\begin{aligned} {[}0,1] = I_1 \cup \cdots \cup I_n \end{aligned}$$

where we put a point $x \in [0,1]$ into $I_i$ if *x* is closer to $\omega _i$ that to any other points in ${\mathcal {N}}$. (We break any ties arbitrarily.)

We can quantize any signed measure $\nu$ on [0, 1] by defining

5.5 $$\begin{aligned} \nu _{\mathcal {N}}\left( \{\omega _i\} \right) = \nu (I_i), \quad i=1,\ldots ,n. \end{aligned}$$

Obviously, $\nu _{\mathcal {N}}$ is a signed measure on ${\mathcal {N}}$. Moreover, if $\nu$ is a measure, then so is $\nu _{\mathcal {N}}$. And if $\nu$ is a probability measure, then so is $\nu _{\mathcal {N}}$. In the latter case, it follows from the construction that

5.6 $$\begin{aligned} W_1(\nu ,\nu _{\mathcal {N}}) \le 1/n. \end{aligned}$$

(By definition of the net, transporting any point *x* to the closest point $\omega _i$ covers distance at most 1/*n*.)

##### Lemma 5.3

(Quantization is a contraction in TV metric) Any signed measure $\nu$ on [0, 1] satisfies

$$\begin{aligned} \Vert {\nu _{\mathcal {N}}} \Vert _\textrm{TV}\le \Vert {\nu } \Vert _\textrm{TV}. \end{aligned}$$

##### Proof

Using (2.5), (5.5), and (2.4), we obtain

$$\begin{aligned} \Vert {\nu _{\mathcal {N}}} \Vert _\textrm{TV}=\frac{1}{2} \sum _{i=1}^n \left| { \nu _{\mathcal {N}}(\{\omega _i\})}\right| = \frac{1}{2} \sum _{i=1}^n \left| {\nu (I_i)}\right| \le \Vert {\nu } \Vert _\textrm{TV}. \end{aligned}$$

The lemma is proved. $\square$

#### A private measure on the interval

##### Theorem 5.4

(Private measure on the interval) Let $\alpha \ge 2$. There exists a randomized algorithm ${\mathcal {A}}$ that takes a probability measure $\mu$ on [0, 1] as an input and returns a finitely-supported probability measure $\nu$ on [0, 1] as an output, and with the following two properties.

(i) (Privacy): the algorithm ${\mathcal {A}}$ is $\alpha$-metrically private in the TV metric.
(ii) (Accuracy): for any input measure $\mu$, the expected accuracy of the output measure $\nu$ in the Wasserstein distance is
  $$\begin{aligned} {{\,\mathrm{{\mathbb {E}}}\,}}W_1 \left( \nu ,\mu \right) \le \frac{C \log ^{\frac{3}{2}} \alpha }{\alpha }. \end{aligned}$$

##### Proof

Take a measure $\mu$ on [0, 1], preprocess it by quantizing as in the previous subsection, and feed the quantized measure $\mu _{\mathcal {N}}$ into the algorithm ${\mathcal {B}}$ of Proposition 5.2 for $\Omega ={\mathcal {N}}$.

The contraction property (Lemma 5.3) ensures that

$$\begin{aligned} \Vert {\mu _{\mathcal {N}}- \mu '_{\mathcal {N}}} \Vert _\textrm{TV}\le \Vert {\mu - \mu '} \Vert _\textrm{TV}. \end{aligned}$$

This and the privacy property of Proposition 5.2 for measures on ${\mathcal {N}}$ guarantee that quantization does not destroy privacy, i.e. the algorithm $\mu \mapsto {\mathcal {B}}(\mu _{\mathcal {N}})$ is still $\alpha$-metrically private as claimed.

As for the accuracy, Proposition 5.2 for the measure $\mu _{\mathcal {N}}$ gives

$$\begin{aligned} {{\,\mathrm{{\mathbb {E}}}\,}}W_1 \left( {\mathcal {B}}(\mu _{\mathcal {N}}), \mu _{\mathcal {N}}\right) \le \frac{C \log ^{\frac{3}{2}} n}{\alpha }. \end{aligned}$$

Moreover, the accuracy of quantization (5.6) states that $W_1(\mu ,\mu _{\mathcal {N}}) \le 1/n$. By triangle inequality, we conclude that

$$\begin{aligned} {{\,\mathrm{{\mathbb {E}}}\,}}W_1 \left( {\mathcal {B}}(\mu _{\mathcal {N}}), \mu \right) \le \frac{1}{n} + \frac{C \log ^{\frac{3}{2}} n}{\alpha }. \end{aligned}$$

Taking *n* to be the largest integer less than or equal to $\alpha$ yields the conclusion of the theorem. $\square$

## The traveling salesman problem

In order to extend the construction of the private measure on the interval [0, 1] to a general metric space $(T,\rho )$, a natural approach would be to map the interval [0, 1] onto some *space-filling curve* of *T*. Since a space filling curves usually are infinitely long, we should do this on the discrete level, for some $\delta$-net of *T* rather than *T* itself. In this section, we will bound length of such discrete space-filling curve in terms of the metric geometry of *T*. In the next section, we will see how this bound determines the accuracy of a private measure in *T*.

A natural framework for this step is related to Traveling Salesman Problem (TSP), which is a central problem in optimization and computer science, and whose history goes back to at least 1832 [6].

Let $G=(V,E)$ be an undirected weighted connected graph. We occasionally refer to the weights of the edges as lengths. A *tour* of *G* is a connected walk on the edges that visits every vertex at least once, and returns to the starting vertex. The TSP is the problem of finding a tour of *G* with the shortest length. Let us denote this length by ${{\,\textrm{TSP}\,}}(G)$.

Although it is NP-hard to compute TSP(G), or even to approximate it within a factor of 123/122 [29], an algorithm of Christofides and Serdyukov [15, 42] from 1976 gives a 3/2-approximation for TSP, and it was shown recently that the factor 3/2 can be further improved [28].

### TSP in terms of the minimum spanning tree

Within a factor of 2, the traveling salesman problem is equivalent to another key problem, namely the problem of finding the *minimum spanning tree* (MST) of *G*. A spanning tree of *G* is a subgraph that is a tree and which includes all vertices of *G*. It always exists and can be found in polynomial time [32, 38]. A spanning tree of *G* with the smallest length is called the minimum spanning tree of *G*; we denote its length by ${{\,\textrm{MST}\,}}(G)$. The following equivalence is a folklore.

#### Lemma 6.1

Any undirected weighted connected graph *G* satisfies

$$\begin{aligned} {{\,\textrm{MST}\,}}(G) \le {{\,\textrm{TSP}\,}}(G) \le 2 {{\,\textrm{MST}\,}}(G). \end{aligned}$$

#### Proof

For the lower bound, it is enough to find a spanning tree of *G* of length bounded by ${{\,\textrm{TSP}\,}}(G)$. Consider the minimal tour of *G* of length ${{\,\textrm{TSP}\,}}(G)$ as a subgraph of *G*. Let *T* be a spanning tree of the tour. Since the tour contains all vertices of *G*, so does *T*, and thus *T* is a spanning tree of *G*. Since *T* is obtained by removing some edges of the tour, the length of *T* is bounded by of the tour, which is ${{\,\textrm{TSP}\,}}(G)$. The lower bound is proved.

**Fig. 3:**
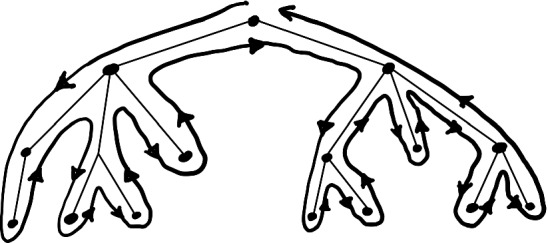
The depth-first search tour demonstrates that the TSP of a tree equals twice the sum of lengths of its edges

For the upper bound, note that dropping any edges of *G* can only increase the value of TSP. Thus TSP of *G* is bounded by the TSP of its spanning tree *T*. Moreover, TSP of any tree *T* equals twice the sum of lengths of the edges of *T*. This can be seen by considering the *depth-first search* tour of *T*, which starts at the root and explores as deep as possible along each branch before backtracking, see Fig. 3. $\square$

### Metric TSP

Let $(T,\rho )$ be a finite metric space. We can consider *T* as a complete weighted graph, whose weights of edges are defined as the distances between the points. The TSP for $(T,\rho )$ is known as *metric TSP*.

Although a tour can visit the same vertex of *T* multiple times, this can be prevented by skipping the vertices previously visited. The triangle inequality shows that skipping can only decrease the length of the tour. Therefore, the shortest tour in a complete graph is always a *Hamiltonian cycle*, a walk that visits all vertices of *T* exactly once before returning to the starting vertex. Let us record this observation:

#### Lemma 6.2

The TSP of a finite metric space $(T,\rho )$ equals the smallest length of a Hamiltonian cycle of *T*.

### A geometric bound on TSP

We would like to compute ${{\,\textrm{TSP}\,}}(T)$ in terms of the geometry of the metric space $(T,\rho )$. Here we will prove an upper bound on ${{\,\textrm{TSP}\,}}(T)$ in terms of the covering numbers. Recall that the *covering number* $N(T,\rho ,\varepsilon )$ is defined as the smallest cardinality of an $\varepsilon$-net of *T*, or equivalently the smallest number of closed balls with centers in *T* and radii $\varepsilon$ whose union covers *T*, see [48, Section 4.2].

#### Theorem 6.3

(TSP via covering numbers) For any finite metric space $(T,\rho )$, we have

$$\begin{aligned} {{\,\textrm{TSP}\,}}(T) \le 16 \int _0^\infty \left( N(T,\rho ,x)-1 \right) \, dx. \end{aligned}$$

#### Proof

*Step 1: constructing a spanning tree.* Let us construct a small spanning tree $T_0$ of *T* and use Lemma 6.1. Let $\varepsilon _j = 2^{-j}$, $j \in {\mathbb {Z}}$, and let ${\mathcal {N}}_j$ be $\varepsilon _j$-nets of *T* with cardinalities $\left| {{\mathcal {N}}_j}\right| = N(T,\rho ,\varepsilon _j)$. Since *T* is finite, we must have $\left| {{\mathcal {N}}_j}\right| = 1$ for all sufficiently small *j*. Let $j_0$ be the largest integer for which $\left| {{\mathcal {N}}_{j_0}}\right| = 1$.

**Fig. 4:**
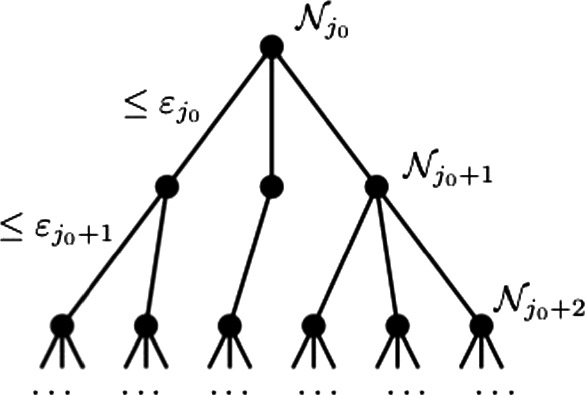
Chaining: construction of a spanning tree of a metric space

At the root of $T_0$, let us put a single point that forms the net ${\mathcal {N}}_{j_0}$. At the next level, put all the points of the net ${\mathcal {N}}_{j_0+1}$, and connect them to the root by edges. The weights of these edges, which are defined as the distances of the points to the root, are all bounded by $\varepsilon _{j_0}$. At the next level, put all points of the net ${\mathcal {N}}_{j_0+2}$, and connect each such point to the closest point in the previous level ${\mathcal {N}}_{j_0+1}$. (Break any ties arbitrarily.) Since the latter set is a $\varepsilon _{j_0+1}$-net, the weights of all these edges are bounded by $\varepsilon _{j_0+1}$. Repeat these steps until the levels do not grow anymore, i.e. until the level contains all the points in *T*; see Fig. 4 for illustration.

If all the nets ${\mathcal {N}}_j$ that make up the levels of the tree $T_0$ are disjoint, then $T_0$ is a spanning tree of *T*. Assume that this is the case for time being.

*Step 2: bounding the length of the tree.* For each of the levels $j = j_0+1, j_0+2,\ldots$, the tree $T_0$ has $\left| {{\mathcal {N}}_j}\right|$ edges connecting the points of level *j* to the level $j-1$, and each such edge has length (weight) bounded by $\varepsilon _{j-1}$. So ${{\,\textrm{MST}\,}}(T)$ is bounded by the sum of the lengths of the edges of $T_0$, i.e.

$$\begin{aligned} {{\,\textrm{MST}\,}}(T) \le \sum _{j=j_0+1}^\infty \varepsilon _{j-1} \left| {{\mathcal {N}}_j}\right| . \end{aligned}$$

*Step 3: bounding the sum by the integral.* Our choice $\varepsilon _j = 2^{-j}$ yields $\varepsilon _{j-1} = 4(\varepsilon _j-\varepsilon _{j+1})$. Moreover, our choice of $j_0$ yields $\left| {{\mathcal {N}}_j}\right| \ge 2$ for all $j \ge j_0+1$, which implies $\left| {{\mathcal {N}}_j}\right| \le 2 \left( \left| {{\mathcal {N}}_j}\right| -1 \right)$ for such *j*. Therefore

6.1 $$\begin{aligned} {{\,\textrm{MST}\,}}(T)&\le 8 \sum _{j=j_0+1}^\infty \left( \varepsilon _j-\varepsilon _{j+1} \right) \left( \left| {{\mathcal {N}}_j}\right| -1 \right) \nonumber \\&\quad = 8 \sum _{j=j_0+1}^\infty \int _{\varepsilon _{j+1}}^{\varepsilon _j} \left( N(T,\rho ,\varepsilon _j)-1 \right) dx \quad \text {(since }\left| {{\mathcal {N}}_j}\right| = N(T,\rho ,\varepsilon _j)\text {)} \nonumber \\&\quad \le 8 \int _0^\infty \left( N(T,\rho ,x)-1 \right) \, dx. \end{aligned}$$

An application of Lemma 6.1 completes the proof.

*Step 4: splitting.* The argument above assumes that all levels ${\mathcal {N}}_j$ of the tree $T_0$ are disjoint. This assumption can be enforced by splitting the points of *T*. If, for example, a point $\omega \in {\mathcal {N}}_j$ is also used in ${\mathcal {N}}_k$ for some $k < j$, add to *T* another a replica of $\omega$ – a point $\omega '$ that has zero distance to $\omega$ and the same distances to all other points as $\omega$. Use $\omega$ in ${\mathcal {N}}_j$ and $\omega '$ in ${\mathcal {N}}_k$. Preprocessing the metric space $(T,\rho )$ by such splitting yields a pseudometric space $(T',\rho )$ in which all levels ${\mathcal {N}}_j$ are disjoint, and whose TSP is the same. $\square$

#### Remark 6.4

(Integrating up to the diameter) Note that $N(T,\rho ,x) = 1$ for any $x>{{\,\textrm{diam}\,}}(T)$, since any single point makes an *x*-net of *T* for such *x*. Therefore, the integrand in Theorem 6.3 vanishes for such *x*, and we have

6.2 $$\begin{aligned} {{\,\textrm{TSP}\,}}(T) \le 16 \int _0^{{{\,\textrm{diam}\,}}(T)} N(T,\rho ,x) \, dx. \end{aligned}$$

### Folding

It is a simple observation that an interval of length ${{\,\textrm{TSP}\,}}(T)$ can be embedded, or “folded”, into *T*:

#### Proposition 6.5

(Folding) For any finite metric space $(T,\rho )$ there exists a finite subset $\Omega$ of the interval $[0,{{\,\textrm{TSP}\,}}(T)]$ and a 1-Lipschitz bijection $F: \Omega \rightarrow T$.

**Fig. 5:**
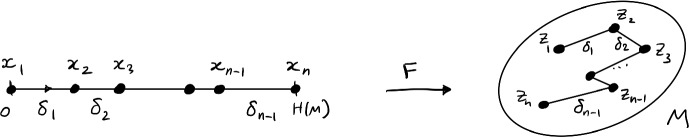
The map *F* folds an interval $[0,{{\,\textrm{TSP}\,}}(M)]$ into a Hamiltonian path (a “space-filling curve”) of the metric space *T*

Heuristically, the map *F* “folds” the interval $[0,{{\,\textrm{TSP}\,}}(T)]$ into the shortest Hamiltonian path of the metric space *T*, see Fig. 5. We can think of this as a *space-filling curve* of *T*.

#### Proof

Let us exploit the heuristic idea of folding. Fix a Hamiltonian cycle in *T* of length ${{\,\textrm{TSP}\,}}(T)$, whose existence is given by Lemma 6.2. Formally, this means that we can label the elements of the space as $T = \{z_1,\ldots ,z_n\}$ in such a way that the lengths

$$\begin{aligned} \delta _i = \rho \left( z_{i+1}, z_i \right) , \quad i=1,\ldots ,n-1, \end{aligned}$$

satisfy $\sum _{i=1}^{n-1} \delta _i \le {{\,\textrm{TSP}\,}}(T)$. Define $\Omega = \{x_1,\ldots ,x_n\}$ by

$$\begin{aligned} x_1=0; \quad x_k = \sum _{i=1}^{k-1} \delta _i, \; k=2,\ldots ,n. \end{aligned}$$

Then all $x_k \le {{\,\textrm{TSP}\,}}(T)$, so $\Omega \subset [0,{{\,\textrm{TSP}\,}}(T)]$ as claimed.

Note that for every $k=1,\ldots ,n-1$ we have

$$\begin{aligned} \rho \left( z_{k+1}, z_k \right) = \delta _k = x_{k+1}-x_k. \end{aligned}$$

Then, for any integers $1 \le k \le k+j \le n$, triangle inequality and telescoping give

$$\begin{aligned} \rho \left( z_{k+j}, z_k \right)&\le \rho \left( z_{k+j}, z_{k+j-1} \right) + \rho \left( z_{k+j-1}, z_{k+j-2} \right) + \cdots + \rho \left( z_{k+1}, z_k \right) \\&= \left( x_{k+j} - x_{k+j-1} \right) + \left( x_{k+j-1} - x_{k+j-2} \right) + \cdots + \left( x_{k+1}- x_k \right) \\&=x_{k+j} - x_k. \end{aligned}$$

This shows that the folding map $F: x_k \mapsto z_k$ is a bijection that satisfies

$$\begin{aligned} \rho \left( F(x), F(y) \right) \le \left| {x-y}\right| \quad \text {for all } x,y \in \Omega . \end{aligned}$$

In other words, *F* is 1-Lipschitz. The proof is complete. $\square$

## A private measure on a metric space

We are ready to construct a private measure on an arbitrary compact metric space $(T,\rho )$. We do this as follows: (a) discretize *T* replacing it with a finite $\delta$-net; (b) fold an interval of length ${{\,\textrm{TSP}\,}}(T)$ onto *T* using Proposition 6.5; and (c) using this folding, pushforward onto *T* the private measure on the interval constructed in Sect. 5. The accuracy of the resulting private measure on *T* is determined by the length of the interval ${{\,\textrm{TSP}\,}}(T)$, which in turn can be expressed using the covering numbers of *T* (Theorem 6.3).

### Finite metric spaces

Let us start by extending Proposition 5.2 from a finite subset on [0, 1] to a finite subset of $(T,\rho )$.

#### Proposition 7.1

(Private measure on a finite metric space) Let $(T,\rho )$ be a finite metric space and let $n = \left| {T}\right|$. Let $\alpha >0$. There exists a randomized algorithm ${\mathcal {B}}$ that takes a probability measure $\mu$ on *T* as an input and returns a probability measure $\nu$ on *T* as an output, and with the following two properties.

(i) (Privacy): the algorithm ${\mathcal {B}}$ is $\alpha$-metrically private in the TV metric.
(ii) (Accuracy): for any input measure $\mu$, the expected accuracy of the output measure $\nu$ in the Wasserstein distance is
  $$\begin{aligned} {{\,\mathrm{{\mathbb {E}}}\,}}W_1(\nu , \mu ) \le \frac{C \log ^{\frac{3}{2}} n}{\alpha } \, {{\,\textrm{TSP}\,}}(T). \end{aligned}$$

#### Proof

Applying Folding Proposition 6.5, we obtain an *n*-element subset $\Omega \subset [0,{{\,\textrm{TSP}\,}}(T)]$ and a 1-Lipschitz bijection $F: \Omega \rightarrow T$. Applying Proposition 5.2 and rescaling by the factor ${{\,\textrm{TSP}\,}}(T)$, we obtain an $\alpha$-metrically private algorithm ${\mathcal {B}}$ that transforms a probability measure $\mu$ on $\Omega$ into a probability measure $\nu$ on $\Omega$, and whose accuracy is

7.1 $$\begin{aligned} {{\,\mathrm{{\mathbb {E}}}\,}}W_1(\nu , \mu ) \le \frac{C \log ^{\frac{3}{2}} n}{\alpha } \, {{\,\textrm{TSP}\,}}(T). \end{aligned}$$

Define a new metric ${\bar{\rho }}$ on $\Omega$ by ${\bar{\rho }}(x,y) = \rho \left( F(x),F(y) \right)$. Since *F* is 1-Lipschitz, we have ${\bar{\rho }}(x,y) \le \left| {x-y}\right|$. Note that the Wasserstein distance can only become smaller if the underlying metric is replaced by a smaller metric. Therefore, the bound (7.1), which holds with respect to the usual metric $\left| {x-y}\right|$ on $\Omega$, automatically holds with respect to the smaller metric ${\bar{\rho }}(x,y)$.

It remains to note that $(\Omega , {\bar{\rho }})$ is isometric to $(T,\rho )$. So the accuracy result (7.1), which as we saw holds in $(\Omega , {\bar{\rho }})$, automatically transfers to $(T,\rho )$ (by considering the pushforward measure). $\square$

### General metric spaces

Quantization allows us to pass from discrete metric spaces to general spaces. A similar technique was used in Sect. 5.2 for the interval [0, 1]. We will repeat it here for a general metric space.

#### Quantization

Fix $\delta > 0$ and let ${\mathcal {N}}= \{\omega _1,\ldots ,\omega _n\}$ be a $\delta$-net of *T* such that $n = \left| {{\mathcal {N}}}\right| = N(T,\rho ,\delta )$. Consider the proximity partition

$$\begin{aligned} T = I_1 \cup \cdots \cup I_n \end{aligned}$$

where we put a point $x \in T$ into $I_i$ if *x* is closer to $\omega _i$ that to any other points in ${\mathcal {N}}$. (We break any ties arbitrarily.)

We can quantize any signed measure $\nu$ on *T* by defining

$$\begin{aligned} \nu _{\mathcal {N}}\left( \{\omega _i\} \right) = \nu (I_i), i=1,\ldots ,n. \end{aligned}$$

Obviously, $\nu _{\mathcal {N}}$ is a signed measure on ${\mathcal {N}}$. Moreover, if $\nu$ is a measure, then so is $\nu _{\mathcal {N}}$. And if $\nu$ is a probability measure, then so is $\nu _{\mathcal {N}}$. In the latter case, it follows from the construction that

7.2 $$\begin{aligned} W_1(\nu ,\nu _{\mathcal {N}}) \le \delta . \end{aligned}$$

(By definition of the net, transporting any point *x* to the closest point $\omega _i$ covers distance at most $\delta$.) Furthermore, Lemma 5.3 easily generalizes and yields

7.3 $$\begin{aligned} \Vert {\nu _{\mathcal {N}}} \Vert _\textrm{TV}\le \Vert {\nu } \Vert _\textrm{TV}. \end{aligned}$$

Finally, let us bound the TSP of the net ${\mathcal {N}}$ using Theorem 6.3. We trivially have $N({\mathcal {N}},\rho ,x) \le \left| {{\mathcal {N}}}\right| = N(T,\rho ,\delta )$ for any $x>0$. Moreover, since ${\mathcal {N}}\subset T$, we also have $N({\mathcal {N}},\rho ,x) \le N(T,\rho ,x/2)$, see [48, Exercise 4.2.10]. Using the former bound for $x<2\delta$ and the latter bound for $x \ge 2\delta$ and applying (6.2), we obtain

7.4 $$\begin{aligned} {{\,\textrm{TSP}\,}}({\mathcal {N}})&\lesssim \int _0^{{{\,\textrm{diam}\,}}({\mathcal {N}})} N({\mathcal {N}},\rho ,x) \, dx \nonumber \\&\le 2\delta N(T,\rho ,\delta ) + \int _{2\delta }^{{{\,\textrm{diam}\,}}(T)} N(T,\rho ,x/2) \, dx \nonumber \\&= 2 \left( \delta N(T,\rho ,\delta ) + \int _\delta ^{{{\,\textrm{diam}\,}}(T)/2} N(T,\rho ,x) \, dx \right) \nonumber \\&\le 2 \left( 2\int _{\delta /2}^{\delta }N(T,\rho ,x)\,dx + \int _\delta ^{{{\,\textrm{diam}\,}}(T)/2} N(T,\rho ,x) \, dx \right) \nonumber \\&\le 4\int _{\delta /2}^{{{\,\textrm{diam}\,}}(T)/2}N(T,\rho ,x)\,dx. \end{aligned}$$

#### A private measure on a general metric space

##### Theorem 7.2

(Private measure on a metric space) Let $(T,\rho )$ be a compact metric space. Let $\alpha , \delta >0$. There exists a randomized algorithm ${\mathcal {A}}$ that takes a probability measure $\mu$ on *T* as an input and returns a finitely-supported probability measure $\nu$ on *T* as an output, and with the following two properties.

(i) (Privacy): the algorithm ${\mathcal {A}}$ is $\alpha$-metrically private in the TV metric.
(ii) (Accuracy): for any input measure $\mu$, the expected accuracy of the output measure $\nu$ in the Wasserstein distance is
  $$\begin{aligned} {{\,\mathrm{{\mathbb {E}}}\,}}W_1(\nu ,\mu ) \le 2\delta + \frac{C}{\alpha } \log ^{\frac{3}{2}} \left( N(T,\rho ,\delta ) \right) \int _{\delta }^{{{\,\textrm{diam}\,}}(T)}N(T,\rho ,x)\,dx. \end{aligned}$$

##### Proof

Preprocess the input measure $\mu$ by quantizing as in the previous subsection, and feed the quantized measure $\mu _{\mathcal {N}}$ into the algorithm ${\mathcal {B}}$ of Proposition 7.1 for the metric space $({\mathcal {N}},\rho )$.

The contraction property (7.3) ensures that

$$\begin{aligned} \Vert {\mu _{\mathcal {N}}- \mu '_{\mathcal {N}}} \Vert _\textrm{TV}\le \Vert {\mu - \mu '} \Vert _\textrm{TV}\end{aligned}$$

for any two input measures $\mu$ and $\mu '$. This and the privacy property in Proposition 7.1 for measures on ${\mathcal {N}}$ guarantee that quantization does not destroy privacy, i.e. the algorithm ${\mathcal {A}}: \mu \mapsto {\mathcal {B}}(\mu _{\mathcal {N}})$ is still $\alpha$-metrically private as claimed.

Next, the accuracy property in Proposition 7.1 for the measure $\mu _{\mathcal {N}}$ on ${\mathcal {N}}$ gives

$$\begin{aligned} {{\,\mathrm{{\mathbb {E}}}\,}}W_1 \left( {\mathcal {B}}(\mu _{\mathcal {N}}), \mu _{\mathcal {N}}\right) \le \frac{C}{\alpha } \log ^{\frac{3}{2}} (N(T,\rho ,\delta )) \, {{\,\textrm{TSP}\,}}({\mathcal {N}}). \end{aligned}$$

Moreover, the accuracy of quantization (7.2) states that $W_1(\mu ,\mu _{\mathcal {N}}) \le \delta$. By triangle inequality, we conclude that

$$\begin{aligned} {{\,\mathrm{{\mathbb {E}}}\,}}W_1 \left( {\mathcal {B}}(\mu _{\mathcal {N}}), \mu \right) \le \delta + \frac{C}{\alpha } \log ^{\frac{3}{2}} (N(T,\rho ,\delta )) \, {{\,\textrm{TSP}\,}}({\mathcal {N}}). \end{aligned}$$

Thus, by (7.4),

$$\begin{aligned} {{\,\mathrm{{\mathbb {E}}}\,}}W_1 \left( {\mathcal {B}}(\mu _{\mathcal {N}}), \mu \right) \le \delta + \frac{C}{\alpha } \log ^{\frac{3}{2}} (N(T,\rho ,\delta )) \, \int _{\delta /2}^{{{\,\textrm{diam}\,}}(T)/2}N(T,\rho ,x)\,dx. \end{aligned}$$

Since $N(T,\rho ,2\delta )\le N(T,\rho ,\delta )$, replacing $\delta$ by $2\delta$ completes the proof of the theorem. $\square$

### Private synthetic data

The output of the algorithm ${\mathcal {A}}$ in Theorem 7.2 is a finitely-supported probability measure $\nu$ on *T*. Quantization allows to transform $\nu$ into an *empirical measure*

7.5 $$\begin{aligned} \mu _Y = \frac{1}{m} \sum _{i=1}^m \delta _{Y_i} \end{aligned}$$

where $Y_1,\ldots ,Y_m$ is some finite sequence of elements of *T*, in which repetitions are allowed. In other words, we can make the output of our algorithm a *synthetic data* $Y=(Y_1,\ldots ,Y_m)$. Let us record this observation.

#### Corollary 7.3

(Outputting an empirical measure) Let $(T,\rho )$ be a compact metric space. Let $\alpha ,\delta >0$. There exists a randomized algorithm ${\mathcal {A}}$ that takes a probability measure $\mu$ on *T* as an input and returns $Y = (Y_1,\ldots ,Y_m) \in T^m$ for some *m* as an output, and with the following two properties.

(i) (Privacy): the algorithm ${\mathcal {A}}$ is $\alpha$-metrically private in the TV metric.
(ii) (Accuracy): for any input measure $\mu$, the expected accuracy of the empirical measure $\mu _Y$ in the Wasserstein distance is
  $$\begin{aligned} {{\,\mathrm{{\mathbb {E}}}\,}}W_1 \left( \mu _Y, \mu \right) \le 3\delta + \frac{C}{\alpha } \log ^{\frac{3}{2}} \left( N(T,\rho ,\delta ) \right) \int _{\delta }^{{{\,\textrm{diam}\,}}(T)}N(T,\rho ,x)\,dx. \end{aligned}$$

#### Proof

Since the output probability measure $\nu$ in Theorem 7.2 is finitely supported, it has the form

$$\begin{aligned} \nu = \sum _{i=1}^r w_i \, \delta _{Y_i} \end{aligned}$$

for some natural number *r*, positive weights $w_i$ and elements $R_i \in T$.

Let us quantize the weights $w_i$ by the uniform quantizer with step 1/*m* where *m* is a large integer. Namely, set

$$\begin{aligned} q(w_i) :=\frac{\lfloor m \, w_i \rfloor }{m}. \end{aligned}$$

Obviously, the total quantization error satisfies

7.6 $$\begin{aligned} \kappa :=\sum _{i=1}^r \left( w_i-q(w_i) \right) \in [0,r/m]. \end{aligned}$$

To make the quantized weights a probability measure, let us add the total quantization error to any given weight, say the first. Thus, define

$$\begin{aligned} w'_1 :=q(w_1) + \kappa \quad \text {and} \quad w'_i :=q(w_i), \; i=2,\ldots ,r \end{aligned}$$

and set

$$\begin{aligned} \nu ' :=\sum _{i=1}^r w'_i \, \delta _{Y_i}. \end{aligned}$$

Note the three key properties of $\nu '$. First, since the weights $w'_i$ sum to one, $\nu '$ is a probability measure. Second, since $\nu '$ is obtained from $\nu$ by transporting a total mass of $\kappa$ across the metric space *T*, we have

$$\begin{aligned} W_1(\nu ,\nu ') \le \kappa \cdot {{\,\textrm{diam}\,}}(T) \le \frac{r}{m} \cdot {{\,\textrm{diam}\,}}(T) \le \delta \end{aligned}$$

where the second inequality follows from (7.6) and the last one by choosing *m* large enough. Third, all quantized weights $q(w_i)$ belong to $\frac{1}{m} {\mathbb {Z}}$ by definition. Thus, $\kappa = 1 - \sum _{i=1}^r q(w_i)$ is also in $\frac{1}{m} {\mathbb {Z}}$. Therefore, all weights $w'_i$ are in $\frac{1}{m} {\mathbb {Z}}$, too. Hence, $w'_i = m_i/m$ for some nonnegative integers $m_i$. In other words,

$$\begin{aligned} \nu ' = \frac{1}{m} \sum _{i=1}^r m_i \, \delta _{Y_i}. \end{aligned}$$

Since $\nu '$ is a probability measure, we must have $\sum _{i=1}^r m_i = m$. Redefine the sequence $Y_1,\ldots ,Y_m$ by repeating each element $Y_i$ of the sequence $Y_1,\ldots ,Y_r$ exactly $m_i$ times. Thus $\nu ' = \frac{1}{m} \sum _{i=1}^m \delta _{Y_i}$, as required. $\square$

Corollary 7.3 allows us to transform any true data $X=(X_1,\ldots ,X_n)$ into a private synthetic data $Y=(Y_1,\ldots ,Y_m)$. To do this, feed the algorithm ${\mathcal {A}}$ with the empirical measure on the true data $\mu _X = \frac{1}{n} \sum _{i=1}^n \delta _{X_i}$. Recall from Lemma 4.1 that if the algorithm ${\mathcal {A}}$ is $\alpha$-metrically private for $\alpha = \varepsilon n$, then the algorithm $X \mapsto Y = {\mathcal {A}}(\mu _X)$ yields $\varepsilon$-differential private synthetic data. Let us record this observation:

#### Corollary 7.4

(Differentially private synthetic data) Let $(T,\rho )$ be a compact metric space. Let $\varepsilon ,\delta >0$. There exists a randomized algorithm ${\mathcal {A}}$ that takes true data $X=(X_1,\ldots ,X_n) \in T^n$ as an input and returns synthetic data $Y = (Y_1,\ldots ,Y_m) \in T^m$ for some *m* as an output, and with the following two properties.

(i) (Privacy): the algorithm ${\mathcal {A}}$ is $\varepsilon$-differentially private.
(ii) (Accuracy): for any true data *X*, the expected accuracy of the synthetic data *Y* is
  $$\begin{aligned} {{\,\mathrm{{\mathbb {E}}}\,}}W_1 \left( \mu _Y, \mu _X \right) \le 3\delta + \frac{C}{\varepsilon n} \log ^{\frac{3}{2}} \left( N(T,\rho ,\delta ) \right) \int _{\delta }^{{{\,\textrm{diam}\,}}(T)}N(T,\rho ,x)\,dx, \end{aligned}$$
  where $\mu _X$ and $\mu _Y$ denote the corresponding empirical measures.

An interested reader may now skip to Sect. 9.1 where we illustrate Corollary 7.4 for a specific example of the metric space, namely the *d*-dimensional cube $T = [0,1]^d$.

#### Remark 7.5

(A polynomial time algorithm) The algorithm in Corollary 7.3 works in *polynomial time with respect to the size* *n* *of the input data, size* *m* *of the output data, and the covering number* $N(T,\rho ,\delta )$. To see this, first, observe that the superregular random walk defined in (3.6) can clearly be implemented in polynomial time. Using this superregular random walk as a noise, we constructed a private signed measure in Proposition 5.1. Thus, the private signed measure in Proposition 5.1 can be implemented in polynomial time. We then project this signed measure to a probability measure to obtain Proposition 5.2. Since this projection is done via a convex optimization, it can be implemented in polynomial time. Note that Proposition 5.2 is in the context where the universe is a finite subset of an interval. In order to extend this to the context of general metric spaces in Theorem 7.2, we first discretize *T* by a $\delta$-net of *T* and then using a traveling salesman path, we reduce the $\delta$-net of *T* into a finite subset of an interval. The bound in Theorem 6.3 and its proof give a polynomial time algorithm to implement the traveling salesman path. In the case $T=[0,1]^{d}$, the traveling salesman can be done via a space-filling curve. Therefore, the algorithm in Theorem 7.2 can be implemented in polynomial time in $N(T,\rho ,\delta )$. Finally the process of turning a probability measure into a synthetic dataset in the proof of Corollary 7.3 (quantization and replication) can clearly be implemented in polynomial time.

In the particular case where *T* is the cube $[0,1]^d$, which we will specialize to in Corollary 9.2, our algorithm runs in time *polynomial in* *m* *and* *n*. This is because with the optimal choice of $\delta$ we make there, the covering number $N(T,\rho ,\delta )$ is polynomial in *n*.

Although the algorithm can be implemented in polynomial time, it is rather convoluted. We will present a streamlined and practical algorithmic implementation of a synthetic data generation method in a forthcoming paper.

## A lower bound

This section is devoted to impossibility results, which yield *lower* bounds on the accuracy of any private measure on a general metric space $(T,\rho )$. While there may be a gap between our upper and lower bounds for general metric spaces, we will see in Sect. 9 that this gap vanishes asymptotically for spaces of Minkowski dimension *d*.

The proof of the lower bound uses the geometric method pioneered by Hardt and Talwar [24]. A lower bound is more convenient to express in terms of packing rather than covering numbers. Recall that the *packing number* ${N_\text {pack}}(T,\rho ,\varepsilon )$ of a compact metric space $(T,\rho )$ is defined as the largest cardinality of an $\varepsilon$-separated subset of *T*. The covering and packing numbers are equivalent up to a factor of 2:

8.1 $$\begin{aligned} {N_\text {pack}}(T,\rho ,2\varepsilon ) \le N(T,\rho ,\varepsilon ) \le {N_\text {pack}}(T,\rho ,\varepsilon ), \end{aligned}$$

see [48, Lemma 4.2.8]. Thus, in all results of this section, packing numbers can be replaced by covering numbers at the cost of changing absolute constants.

### A master lower bound

We first prove a general result that establishes limitations of metric privacy. To understand this statement better, it may be helpful to assume that ${\mathcal {M}}_0={\mathcal {M}}_1$ and $\rho _0=\rho _1$ in the first reading.

#### Proposition 8.1

(A master lower bound) Let ${\mathcal {M}}_0 \subset {\mathcal {M}}_1$ be two subsets, and let $\rho _i$ be a metric on ${\mathcal {M}}_i$, $i=0,1$. Assume that for some $t, \alpha > 0$ we have

$$\begin{aligned} {{\,\textrm{diam}\,}}({\mathcal {M}}_0, \rho _0) \le 1 \quad \text {and} \quad {N_\text {pack}}({\mathcal {M}}_0, \rho _1,t) > 2e^\alpha . \end{aligned}$$

Then, for any randomized algorithm ${\mathcal {A}}: {\mathcal {M}}_0 \rightarrow {\mathcal {M}}_1$ that is $\alpha$-metrically private with respect to the metric $\rho _0$, there exists $x \in {\mathcal {M}}_0$ such that

$$\begin{aligned} {{\,\mathrm{{\mathbb {E}}}\,}}\rho _1 \left( {\mathcal {A}}(x), x \right) > t/4. \end{aligned}$$

#### Proof

For contradiction, assume that

8.2 $$\begin{aligned} {{\,\mathrm{{\mathbb {E}}}\,}}\rho _1 \left( {\mathcal {A}}(x), x \right) \le t/4, \end{aligned}$$

for all $x\in {\mathcal {M}}_0$. Let ${\mathcal {N}}$ be a *t*-separated subset of the metric space $({\mathcal {M}}_0, \rho _1)$ with cardinality

8.3 $$\begin{aligned} \left| {{\mathcal {N}}}\right| > 2e^\alpha . \end{aligned}$$

The separation condition implies that the balls $B(y,\rho _1, t/2)$ centered at the points $y \in {\mathcal {N}}$ and with radii *t*/2 are all disjoint.

Fix any reference point $y \in {\mathcal {M}}_0$. The disjointness of the balls yields8.4On the other hand, by the definition of $\alpha$-metric privacy, for each $x \in {\mathcal {N}}$ we have:The diameter assumption yields $\rho _0(x,y) \le 1$. Furthermore, using the assumption (8.2) and Markov’s inequality, we obtainCombining the two bounds givesSubstitute this into (8.4) to get

$$\begin{aligned} \sum _{x \in {\mathcal {N}}} \frac{1}{2e^\alpha } \le 1. \end{aligned}$$

In other words, we conclude that $\left| {{\mathcal {N}}}\right| \le 2e^\alpha$, which contradicts (8.3). The proof is complete. $\square$

Can the compactness assumption of the underlying metric space $(T,\rho )$ be dropped? The covering number of a general non-compact metric space of $(T,\rho )$ is infinite. Hence, our lower bound in Proposition 8.1 shows that it is impossible to establish differential privacy with any accuracy gurantees in that case without imposing additional assumptions.

### Metric entropy of the space of probability measures

For a given compact metric space $(T,\rho )$, we denote by ${\mathcal {M}}(T)$ the collection of all Borel probability measures on *T*. We are going to apply Proposition 8.1 for ${\mathcal {M}}_0 = {\mathcal {M}}_1 = {\mathcal {M}}(T)$, for $\rho _1=$ Wasserstein metric and $\rho _0=$ TV metric. That proposition requires a lower bound on the packing number ${N_\text {pack}}\left( {\mathcal {M}}(T), W_1, t/3 \right)$. In the next lemma, we relate this packing number to that of $(T,\rho )$. Essentially, it says that if *T* is large, then there are a lot of probability measures on *T*.

#### Proposition 8.2

(Metric entropy of the space of probability measures) For any compact metric space $(T,\rho )$ and every $t>0$, we have

$$\begin{aligned} {N_\text {pack}}\left( {\mathcal {M}}(T), W_1, t/3 \right) \ge \exp \left( c {N_\text {pack}}(T,\rho ,t) \right) , \end{aligned}$$

where $c>0$ is a universal constant. 3

The proof will use the following lemma.

#### Lemma 8.3

(A lower bound on the Wasserstein distance) Let $(T,\rho )$ be a *t*-separated 4 compact metric space. Then, for any pair of probability measures $\mu , \nu$ on *T*, we have

$$\begin{aligned} W_1(\mu ,\nu ) \ge \mu (B^c) \, t \quad \text {where} \quad B = {{\,\textrm{supp}\,}}(\nu ). \end{aligned}$$

#### Proof

Suppose that $\gamma$ is a coupling of $\mu$ and $\nu$. Since $\nu$ is supported on *B*, we have $\gamma (B^c \times B^c) \le \gamma (T \times B^c) = \nu (B^c) = 0$, which means that $\gamma (B^c \times B^c)=0$. Therefore

$$\begin{aligned} \gamma (B^c \times B) = \gamma (B^c \times T) - \gamma (B^c \times B^c) = \mu (B^c). \end{aligned}$$

Since the sets $B^c$ and *B* are disjoint, the separation assumption implies that $\rho (x,y) > t$ for all pairs $x\in B^{c}$ and $y\in B$. Thus,

$$\begin{aligned} \int _{T\times T}\rho (x,y)\;d\gamma (x,y)\ge \int _{B^c \times B} \rho (x,y) \; d\gamma (x,y)\ge t\gamma (B^c \times B)=t\mu (B^{c}). \end{aligned}$$

Since this holds for all coupling $\gamma$ of $\mu$ and $\nu$, the result follows. $\square$

#### Lemma 8.4

(Many different measures) Let $({\mathcal {N}},\rho )$ be a *t*-separated compact metric space, and assume that $\left| {{\mathcal {N}}}\right| \ge 2n$ for some $n \in {\mathbb {N}}$. Then there exists a family of at least $\exp (cn)$ empirical measures on *n* points of *T* that are pairwise *t*/3-separated in the Wasserstein distance, where $c>0$ is a universal constant.

#### Proof

Let $\mu = n^{-1} \sum _{i=1}^n \delta _{X_i}$ and $\nu = n^{-1} \sum _{i=1}^n \delta _{Y_i}$ be two independent random empirical measures on *T*. Let us condition on $\nu$ and denote $B = {{\,\textrm{supp}\,}}(\nu )$. Then

$$\begin{aligned} \mu (B^c) = \frac{1}{n} \sum _{i=1}^n {{{\textbf {1}}}}_{\{X_i \in B^c\}}. \end{aligned}$$

Now, ${{{\textbf {1}}}}_{\{X_i \in B^c\}}$ are i.i.d. Bernoulli random variables that take value 1 with probabilitysince by construction we have $\left| {B}\right| \le n$ and by assumption $\left| {{\mathcal {N}}}\right| \ge 2n$. Then, applying Chernoff inequality (see [48, Exercise 2.3.2]), we conclude that $\mu (B^c) > 1/3$ with probability bigger than $1-e^{-5cn}$, where $c>0$ is a universal constant. Lemma 8.3 yields that $W_1(\mu ,\nu ) > t/3$.

Now consider a sequence $\mu _1,\ldots ,\mu _K$ of independent random empirical measures on *T*. Using the result above and taking a union bound we conclude that, with probability at least $$1-\left( {\begin{array}{c}K\\ 2\end{array}}\right) e^{-5cn}$$, the inequality $W_1(\mu _i,\mu _j) > t/3$ holds for all pairs of distinct indices $i,j \in \{1,\ldots ,K\}$. Choosing $K = \lceil e^{cn} \rceil$ makes *K* between $e^{cn}$ (as claimed) and $e^{2cn}$. Thus, the success probability is more than $1- (e^{2cn})^2 e^{-5cn}$, which is positive. The existence of the required family of measures follows. $\square$

#### Proof of Proposition 8.2

Let ${\mathcal {N}}\subset T$ be a *t*-separated subset of cardinality $\left| {{\mathcal {N}}}\right| = {N_\text {pack}}(T,\rho ,t)$. Lemma 8.4 implies the existence of a set of at least $\exp (c \left| {{\mathcal {N}}}\right| )$ probability measures on *T* that is (*t*/3)-separated in the Wasserstein distance. In other words, we have ${N_\text {pack}}\left( {\mathcal {M}}(T), W_1, t/3 \right) \ge \exp (c \left| {{\mathcal {N}}}\right| )$. Proposition 8.2 is proved. $\square$

### Lower bounds for private measures and synthetic data

Now we are ready to prove the two main lower bounds on the accuracy for (a) metrically private measures and (b) differential private data.

#### Theorem 8.5

(Private measure: a lower bound) Let $(T,\rho )$ be a compact metric space. Assume that for some $t>0$ and $\alpha \ge 1$ we have

$$\begin{aligned} {N_\text {pack}}(T, \rho , t) > C\alpha . \end{aligned}$$

Then, for any randomized algorithm ${\mathcal {A}}$ that takes a probability measure $\mu$ on *T* as an input and returns a probability measure $\nu$ on *T* as an output and that is $\alpha$-metrically private with respect to the TV metric, there exists $\mu$ such that

$$\begin{aligned} {{\,\mathrm{{\mathbb {E}}}\,}}W_1(\nu ,\mu ) > t/12. \end{aligned}$$

#### Proof

The assumption on the packing number for a sufficiently large constant *C* and Proposition 8.2 yield

$$\begin{aligned} {N_\text {pack}}\left( {\mathcal {M}}(T), W_1, t/3 \right) \ge e^{2\alpha } > 2e^\alpha . \end{aligned}$$

Next, apply Proposition 8.1 with *t*/3 instead of *t*, and for ${\mathcal {M}}_0 = {\mathcal {M}}_1 = {\mathcal {M}}(T)$, setting $\rho _1$ and $\rho _0$ to be the Wasserstein and the TV metrics, respectively. The required conclusion follows. $\square$

#### Theorem 8.6

(Synthetic data: a lower bound) There exists an absolute constant $n_0$ such that the following holds. Let $(T,\rho )$ be a compact metric space. Assume that for some $t>0$ and and some integer $n>n_0$ we have

$$\begin{aligned} {N_\text {pack}}(T, \rho , t) > 2n. \end{aligned}$$

Then, for any *c*-differentially private randomized algorithm ${\mathcal {A}}$ that takes true data $X=(X_1,\ldots ,X_n) \in T^n$ as an input and returns synthetic data $Y=(Y_1,\ldots ,Y_m) \in T^m$ for some *m* as an output, there exists input data *X* such that

$$\begin{aligned} {{\,\mathrm{{\mathbb {E}}}\,}}W_1(\mu _Y,\mu _X) > t/12, \end{aligned}$$

where $\mu _X$ and $\mu _Y$ denote the empirical measures on *X* and *Y*.

#### Proof

First note that a version of Proposition 8.2 holds for empirical measures. Namely, denote the set of all empirical measures on *n* points of *T* by ${\mathcal {M}}_n(T)$. If ${N_\text {pack}}(T,\rho ,t) > 2n$ then we claim that

8.5 $$\begin{aligned} {N_\text {pack}}\left( {\mathcal {M}}_n(T), W_1, t/3 \right) > 2e^{c_1n}. \end{aligned}$$

To see this, let ${\mathcal {N}}\subset T$ be a *t*-separated subset of cardinality $\left| {{\mathcal {N}}}\right| > 2n$. Lemma 8.4 implies the existence of a set of at least $e^{cn} \ge 2e^{c_1n}$ members of ${\mathcal {M}}_n(T)$ that is (*t*/3)-separated in the Wasserstein distance. The claim (8.5) follows.

In preparation to apply Proposition 8.1, consider the sets ${\mathcal {M}}_0 :=T^n$ and ${\mathcal {M}}_1 :=\cup _{k=1}^\infty T^k$. Consider the normalized Hamming metric

$$\begin{aligned} \rho _0(X,X') = \frac{1}{n} \left| {\{i \in [n]:\; X_i \ne X'_i\}}\right| \end{aligned}$$

on ${\mathcal {M}}_0$, and the Wasserstein metric

$$\begin{aligned} \rho _1(X,X') = W_1(\mu _X,\mu _{X'}) \end{aligned}$$

on ${\mathcal {M}}_1$. Then we clearly have ${{\,\textrm{diam}\,}}({\mathcal {M}}_0,\rho _0) \le 1$, and (8.5) is equivalent to ${N_\text {pack}}({\mathcal {M}}_0,\rho _1,t/3) > 2e^{c_1 n}$.

If ${\mathcal {A}}: {\mathcal {M}}_0 \rightarrow {\mathcal {M}}_1$ is a *c*-differentially private algorithm, then ${\mathcal {A}}$ is (*cn*)-metrically private in the metric $\rho _0$ due to Lemma 2.3. Applying Proposition 8.1 with *t*/3 instead of *t* and $\alpha = c_1n$, we obtain the required conclusion. $\square$

## Examples and asymptotics

### A private measure on the unit cube

Let us work out the bound of Theorem 7.2 for a concrete example: the *d*-dimensional unit cube equipped with the $\ell ^\infty$ metric, i.e. $(T,\rho ) = ([0,1]^d, \, \Vert {\cdot } \Vert _\infty )$. The covering numbers satisfy

$$\begin{aligned} N(T,\Vert {\cdot } \Vert _\infty ,x) \le (1/x)^d, \quad x>0, \end{aligned}$$

since the set $x {\mathbb {Z}}^d \cap [0,1)^d$ forms an *x*-net of *T*. Thus the accuracy is

$$\begin{aligned} {{\,\mathrm{{\mathbb {E}}}\,}}W_1(\nu ,\mu ) \lesssim \delta + \frac{\log ^{\frac{3}{2}} (1/\delta )}{\alpha } \int _\delta ^1 (1/x)^d \, dx \lesssim \delta + \frac{\log ^{\frac{3}{2}} (1/\delta )}{\alpha } \cdot (1/\delta )^{d-1} \end{aligned}$$

if $d \ge 2$. Optimizing in $\delta$ yields

$$\begin{aligned} {{\,\mathrm{{\mathbb {E}}}\,}}W_1(\nu ,\mu ) \lesssim \Big ( \frac{\log ^{\frac{3}{2}} \alpha }{\alpha } \Big )^{1/d}, \end{aligned}$$

which wonderfully extends Theorem 5.4 for $d=1$. Combining the two results, for $d=1$ and $d \ge 2$, we obtain the following general result:

#### Corollary 9.1

(Private measure on the cube) Let $d \in {\mathbb {N}}$ and $\alpha \ge 2$. There exists a randomized algorithm ${\mathcal {A}}$ that takes a probability measure $\mu$ on $[0,1]^d$ as an input and returns a finitely-supported probability measure $\nu$ on $[0,1]^d$ as an output, and with the following two properties.

(i) (Privacy): the algorithm ${\mathcal {A}}$ is $\alpha$-metrically private in the TV metric.
(ii) (Accuracy): for any input measure $\mu$, the expected accuracy of the output measure $\nu$ in the Wasserstein distance is
  $$\begin{aligned} {{\,\mathrm{{\mathbb {E}}}\,}}W_1(\nu ,\mu ) \le C \Big ( \frac{\log ^{\frac{3}{2}} \alpha }{\alpha } \Big )^{1/d}. \end{aligned}$$

Similarly, by invoking Corollary 7.4, we obtain $\varepsilon$-differential privacy for synthetic data:

#### Corollary 9.2

(Private synthetic data in the cube) Let $d,n \in {\mathbb {N}}$ and $\varepsilon >0$. There exists a randomized algorithm ${\mathcal {A}}$ that takes true data $X=(X_1,\ldots ,X_n) \in ([0,1]^d)^n$ as an input and returns synthetic data $Y = (Y_1,\ldots ,Y_m) \in ([0,1]^d)^m$ for some *m* as an output, and with the following two properties.

(i) (Privacy): the algorithm ${\mathcal {A}}$ is $\varepsilon$-differentially private.
(ii) (Accuracy): for any true data *X*, the expected accuracy of the synthetic data *Y* is
  $$\begin{aligned} {{\,\mathrm{{\mathbb {E}}}\,}}W_1 \left( \mu _Y, \mu _X \right) \le C \Big ( \frac{\log ^{\frac{3}{2}} (\varepsilon n)}{\varepsilon n} \Big )^{1/d}, \end{aligned}$$
  where $\mu _X$ and $\mu _Y$ denote the corresponding empirical measures.

The two results above are nearly sharp in the setting when $\varepsilon$ is a constant function of *n* and $n \rightarrow \infty$. Indeed, let us work out the lower bound for the cube, using Theorem 8.5. The covering numbers satisfy

$$\begin{aligned} {N_\text {pack}}(T,\Vert {\cdot } \Vert _\infty ,x) \ge (c/x)^d, \quad x>0, \end{aligned}$$

which again can be seen by considering a rescaled integer grid. Setting $t = c/(2C\alpha )^{1/d}$ we get $N(T,\Vert {\cdot } \Vert _\infty ,t) > C\alpha$. Hence

$$\begin{aligned} {{\,\mathrm{{\mathbb {E}}}\,}}W_1(\nu ,\mu ) > t/12 > rsim (1/\alpha )^{1/d}, \end{aligned}$$

which matches the upper bound in Corollary 9.1 up to a logarithmic factor. Let us record this result.

#### Corollary 9.3

(Private measure on the cube: a lower bound) Let $d \in {\mathbb {N}}$ and $\alpha \ge 2$. Then, for any randomized algorithm ${\mathcal {A}}$ that takes a probability measure $\mu$ on $[0,1]^d$ as an input and returns a probability measure $\nu$ on $[0,1]^d$ as an output, and that is $\alpha$-metrically private with respect to the TV metric, there exists $\mu$ such that

$$\begin{aligned} {{\,\mathrm{{\mathbb {E}}}\,}}W_1(\nu ,\mu ) > c \Big (\frac{1}{\alpha } \Big )^{1/d}. \end{aligned}$$

In a similar way, by invoking the lower bound in Theorem 8.6, we obtain the following nearly matching lower bound for Corollary 9.2:

#### Corollary 9.4

(Private synthetic data in the cube: a lower bound) Let $d,n \in {\mathbb {N}}$. Then, for any *c*-differentially private randomized algorithm ${\mathcal {A}}$ that takes true data $X=(X_1,\ldots ,X_n) \in ([0,1]^d)^n$ as an input and returns synthetic data $Y = (Y_1,\ldots ,Y_m) \in ([0,1]^d)^m$ for some *m* as an output, there exists input data *X* such that

$$\begin{aligned} {{\,\mathrm{{\mathbb {E}}}\,}}W_1(\nu _Y,\mu _X) > c \Big (\frac{1}{n} \Big )^{1/d}. \end{aligned}$$

where $\mu _X$ and $\mu _Y$ denotes the empirical measures on *X* and *Y*.

#### Remark 9.5

(Low dimensions) As we can see, the accuracy bound $n^{-1/d}$ gets worse with increasing dimension *d*, and becomes constant for $d \gg \log n$. Thus, results like Corollary 9.2 are only useful for low dimensions. This should not come as a surprise. As we know from the previously mentioned no-go result by Ullman and Vadhan [44], it is computationally not feasible to construct private synthetic data in high dimensions that accurately preserves even two-way marginals, let alone all Lipschitz queries (which is what Wasserstein metric does).

### Asymptotic result

The only property of the cube $T = [0,1]^d$ we used in the previous section is the behavior on its covering numbers,^5^ namely that

9.1 $$\begin{aligned} N(T,\rho ,x) \asymp (1/x)^{-d}, \quad x>0. \end{aligned}$$

Therefore, the same results on private measures and synthetic data hold for any compact metric space $(T,\rho )$ whose covering numbers behave this way. In particular, it follows that any probability measure $\mu$ on *T* can be transformed into a $\alpha$-metrically private measure $\nu$ on *T*, with accuracy

9.2 $$\begin{aligned} {{\,\mathrm{{\mathbb {E}}}\,}}W(\nu , \mu ) \asymp (1/\alpha )^{1/d}. \end{aligned}$$

(ignoring logarithmic factors), and this result is nearly sharp. Similarly, any true data $X \in T^n$ can be transformed into $\varepsilon$-differentially private synthetic data $Y \in T^m$ for some *m*, with accuracy

9.3 $$\begin{aligned} {{\,\mathrm{{\mathbb {E}}}\,}}W(\mu _Y, \mu _X) \asymp (1/n)^{1/d}. \end{aligned}$$

(ignoring logarithmic factors and dependence on $\varepsilon$), and this result is nearly sharp.

These intuitive observations can be formalized using the notion of *Minkowski dimension*. By definition, the metric space $(T,\rho )$ has Minkowski dimension *d* if

$$\begin{aligned} \lim _{x \rightarrow 0} \frac{\log N(T,\rho ,x)}{\log (1/x)} = d. \end{aligned}$$

The following two asymptotic results combine upper and lower bounds, and essentially show that (9.2) and (9.3) hold in any space of dimension *d*.

#### Theorem 9.6

(Private measure, asymptotically) Let $(T,\rho )$ be a compact metric space of Minkowski dimension $d\ge 1$. Then

$$\begin{aligned} \lim _{\alpha \rightarrow \infty } \inf _{\mathcal {A}}\sup _\mu \frac{\log ( {{\,\mathrm{{\mathbb {E}}}\,}}W_1({\mathcal {A}}(\mu ), \mu ) )}{\log \alpha } = -\frac{1}{d}. \end{aligned}$$

Here the infimum is over randomized algorithms ${\mathcal {A}}$ that input and output a probability measure on *T* and are $\alpha$-metrically private with respect to the TV metric; the supremum is over all probability measures $\mu$ on *T*.

#### Proof

We deduce the upper bound from Theorem 7.2 and the lower bound from Theorem 8.5.

*Upper bound.* By rescaling, we can assume without loss of generality that ${{\,\textrm{diam}\,}}(T,\rho ) = 1$. Fix any $\varepsilon >0$. By definition of Minkowski dimension, there exists $\delta _0>0$ such that

9.4 $$\begin{aligned} N(T,\rho ,x) \le (1/x)^{d+\varepsilon } \quad \text {for all } x \in (0,\delta _0). \end{aligned}$$

Then

$$\begin{aligned} \int _\delta ^1 N(T,\rho ,x) \, dx \le \int _\delta ^{\delta _0} (1/x)^{d+\varepsilon } \, dx + \int _{\delta _0}^1 N(T,\rho ,x) \, dx \le K(1/\delta )^{d+\varepsilon -1} + I(\delta _0) \end{aligned}$$

where $K = 1/(d+\varepsilon -1)$ and $I(\delta _0) = \int _{\delta _0}^1 N(T,\rho ,x) \, dx$. The last step follows if we replace $\delta _0$ by infinity and compute the integral.

If we let $\delta \downarrow 0$, we see that $K(1/\delta )^{d+\varepsilon -1} \rightarrow \infty$ while $I(\delta _0)$ stays the same since it does not depend on $\delta$. Therefore, there exists $\delta _1>0$ such that $I(\delta _0) \le K(1/\delta )^{d+\varepsilon -1}$ for all $\delta \in (0,\delta _1)$. Therefore,

$$\begin{aligned} \int _\delta ^1 N(T,\rho ,x) \, dx \le 2K(1/\delta )^{d+\varepsilon -1} \quad \text {for all } \delta \in (0,\min (\delta _0,\delta _1)). \end{aligned}$$

Applying Theorem 7.2 for such $\delta$ and using (9.4), we get

9.5 $$\begin{aligned} \inf _{\mathcal {A}}\sup _\mu {{\,\mathrm{{\mathbb {E}}}\,}}W_1(\nu ,\mu ) \le 2\delta + \frac{C}{\alpha } \log ^{\frac{3}{2}} \left( (1/\delta )^{d+\varepsilon } \right) \cdot 2K(1/\delta )^{d+\varepsilon -1}. \end{aligned}$$

Optimizing in $\delta$, we find that a good choice is

$$\begin{aligned} \delta = \delta (\alpha ) = \left( \frac{\log ^{\frac{3}{2}}(K\alpha )}{K\alpha } \right) ^{\frac{1}{d+\varepsilon }}. \end{aligned}$$

For any sufficiently large $\alpha$, we have $\delta < \min (\delta _0,\delta _1)$ as required, and substituting $\delta = \delta (\alpha )$ into the bound in (9.5) we get after simplification:

$$\begin{aligned} \inf _{\mathcal {A}}\sup _\mu {{\,\mathrm{{\mathbb {E}}}\,}}W_1(\nu ,\mu ) \le (1+2CK)\delta (\alpha ). \end{aligned}$$

Furthermore, recalling that *K* does not depend on $\alpha$, it is clear that

$$\begin{aligned} \lim _{\alpha \rightarrow \infty } \frac{\log \left( (1+2CK)\delta (\alpha ) \right) }{\log \alpha } = -\frac{1}{d+\varepsilon }. \end{aligned}$$

Thus

$$\begin{aligned} \limsup _{\alpha \rightarrow \infty } \frac{\log ( \inf _{\mathcal {A}}\sup _\mu {{\,\mathrm{{\mathbb {E}}}\,}}W_1(\nu ,\mu ) )}{\log \alpha } \le -\frac{1}{d+\varepsilon }. \end{aligned}$$

Since $\varepsilon >0$ is arbitrary, it follows that

9.6 $$\begin{aligned} \limsup _{\alpha \rightarrow \infty } \inf _{\mathcal {A}}\sup _\mu \frac{\log ( {{\,\mathrm{{\mathbb {E}}}\,}}W_1({\mathcal {A}}(\mu ), \mu ) )}{\log \alpha } \le -\frac{1}{d}. \end{aligned}$$

*Lower bound.* Fix any $\varepsilon >0$. By definition of Minkowski dimension and the equivalence (8.1), there exists $\delta _0>0$ such that

$$\begin{aligned} {N_\text {pack}}(T,\rho ,x) \ge N(T,\rho ,x) > (1/x)^{d-\varepsilon } \quad \text {for all } x \in (0,\delta _0). \end{aligned}$$

Set

$$\begin{aligned} x(\alpha ) = \left( \frac{1}{C\alpha } \right) ^{\frac{1}{d-\varepsilon }}. \end{aligned}$$

Then, for any sufficiently large $\alpha$, we have $x \in (0,\delta _0)$ and

$$\begin{aligned} {N_\text {pack}}(T,\rho ,x(\alpha )) > C\alpha . \end{aligned}$$

Applying Theorem 8.5, we get

$$\begin{aligned} \inf _{\mathcal {A}}\sup _\mu {{\,\mathrm{{\mathbb {E}}}\,}}W_1(\nu ,\mu ) \ge x(\alpha )/20. \end{aligned}$$

It is easy to check that

$$\begin{aligned} \lim _{\alpha \rightarrow \infty } \frac{\log \left( x(\alpha )/20 \right) }{\log \alpha } = -\frac{1}{d-\varepsilon }. \end{aligned}$$

Thus

$$\begin{aligned} \liminf _{\alpha \rightarrow \infty } \frac{\log ( \inf _{\mathcal {A}}\sup _\mu {{\,\mathrm{{\mathbb {E}}}\,}}W_1(\nu ,\mu ) )}{\log \alpha } \ge -\frac{1}{d-\varepsilon }. \end{aligned}$$

Since $\varepsilon >0$ is arbitrary, it follows that

$$\begin{aligned} \liminf _{\alpha \rightarrow \infty } \inf _{\mathcal {A}}\sup _\mu \frac{\log ( {{\,\mathrm{{\mathbb {E}}}\,}}W_1({\mathcal {A}}(\mu ), \mu ) )}{\log \alpha } \ge -\frac{1}{d}. \end{aligned}$$

Combining with the upper bound (9.6), we complete the proof. $\square$

In a similar way, we can deduce the following asymptotic result for private synthetic data. The argument is analogous; the upper bound follows from Corollary 7.4 and the lower bound from Theorem 8.6.

#### Theorem 9.7

Let $(T,\rho )$ be a compact metric space of Minkowski dimension $d\ge 1$. Then, for every $\varepsilon \in (0,c)$, we have

9.7 $$\begin{aligned} \lim _{n \rightarrow \infty } \inf _{\mathcal {A}}\sup _X \frac{\log ( {{\,\mathrm{{\mathbb {E}}}\,}}W_1(\mu _Y, \mu _X) )}{\log n} = -\frac{1}{d}. \end{aligned}$$

Here the infimum is over $\varepsilon$-differentially private randomized algorithms ${\mathcal {A}}$ that take true data $X=(X_1,\ldots ,X_n) \in T^n$ as an input and return synthetic data $Y = {\mathcal {A}}(X) = (Y_1,\ldots ,Y_m) \in T^m$ for some *m* as an output.

#### Remark 9.8

(Low-dimensional data in high dimensions?) This and other results proved here show that the accuracy of private synthetic data must deteriorate quickly as the data dimension *d* increases. But does this mean that the proposed method is useless for any high-dimensional data? In practice, this is not necessarily the case. Real-world high-dimensional data often live in (or near) a low-dimensional smooth manifold. Since a smooth manifold is metrizable and a smooth *d*-dimensional manifold in ${\mathbb {R}}^n$ has Minkowski dimension *d*, our framework may still apply to the standard setting of high-dimensional statistics, where the data lives in high dimension but its intrinsic geometry is low-dimensional. Thus, the generalization via Minkowski dimension presented in this section may not only be appealing from a theoretical viewpoint, but carries a practical potential, which we hope to pursue in our future work.

## Footnotes

1 The factor 2 is chosen for convenience.

2 A Laplacian random variable $X \sim {{\,\textrm{Lap}\,}}(\lambda )$ is defined by , $t \ge 0$. The Laplacian distribution has density $(1/2\lambda ) \exp (-\left| {x}\right| /\lambda )$ on ${\mathbb {R}}$. The mean equals zero and the variance equals $2\lambda ^2$.

3 Throughout Sects. 8 and 9, *c* will always denote the same constant, while the constant *C* may take on different values in different computations.

4 This means that the distance between any two distinct points in *T* is larger than *t*.

5 The lower bound used packing numbers, but they are equivalent to covering numbers due to (8.1).
